# Unsupervised Hierarchical Symbolic Regression for Interpretable Property Modeling in Complex Multi‐Variable Systems

**DOI:** 10.1002/advs.202521200

**Published:** 2026-01-07

**Authors:** Siyu Lou, Chengchun Liu, Dongxiao Zhang, Yuntian Chen, Fanyang Mo

**Affiliations:** ^1^ School of computer science Shanghai Jiao Tong University Shanghai P.R. China; ^2^ Ningbo Key Laboratory of Advanced Manufacturing Simulation Eastern Institute of Technology Ningbo P.R. China; ^3^ School of Materials Science and Engineering Peking University Beijing P.R. China; ^4^ School of Advanced Materials Peking University Shenzhen Graduate School Shenzhen P.R. China; ^5^ AI for Science (AI4S)‐Preferred Program Peking University Shenzhen Graduate School Shenzhen P.R. China; ^6^ Guangdong Provincial Key Laboratory of Nano‐Micro Materials Research Peking University Shenzhen Graduate School Shenzhen P.R. China

**Keywords:** explainable AI, molecular polarity, molecular structure, symbolic regression, TLC

## Abstract

Recent advances in artificial intelligence have produced powerful predictive models for chemical analysis, but model interpretability remains a challenge. Here, we introduce Unsupervised Hierarchical Symbolic Regression (UHSR), providing an explainable solution while maintaining competitive predictive performance. With a focus on thin‐layer chromatography (TLC), a crucial technique in molecular polarity analysis, UHSR automatically distills chemically‐intuitive retention indices and discovers explainable equations that link molecular structures to chromatographic behavior. Experiments have shown UHSR's capability to derive concise and accurate governing equations linking polarity to molecular structures from the TLC dataset. A survey of 100 expert chemists demonstrates that our UHSR model gains more trust from chemists compared to traditional models. Additionally, we also show its adaptability to other property prediction tasks beyond molecular polarity.

## Graphical abstract

UHSR translates complex chemical behavior into clear and explainable equations. Applied to thin‐layer chromatography, it automatically uncovers the mathematical rules linking a molecule's structure to its polarity. This approach matches the accuracy of advanced AI while providing interpretable results, earning greater trust from chemists. The method is also adaptable to diverse property prediction tasks beyond chromatography.

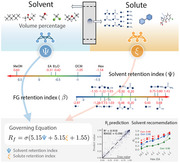

## 1 Introduction

Scientific discoveries aim to explore and understand the world through the observation of phenomena, the systematic collection of data, the identification of patterns, and the elucidation of underlying principles that enable the prediction of future events [1, 2]. The derivation of empirical equations and parameters through data‐driven methods has been pivotal in elucidating chemical phenomena and predicting experimental outcomes. For example, Henry's Law ($C=k_{H}\cdot P$) for solubility predictions [3], the Arrhenius equation ($k=A\cdot e^{-\frac{E_{a}}{RT}}$) for reaction rate dependence on temperature [4], and the van't Hoff equation ($\ln K=-\frac{\Delta H^{\circ }}{RT}+\frac{\Delta S^{\circ }}{R}$) for equilibrium constants related to temperature [5]. These empirical equations and parameters, derived through extensive experimental work and large datasets, remain foundational in contemporary chemistry.

Polarity, reflecting the uniformity and symmetry of charge distributions in molecules, is crucial for understanding molecular interactions and characterizing molecules [6, 7]. Chromatography serves as an essential tool for studying polarity by providing insights into how molecules interact under various chemical environments. Over the years, various chromatographic models have been developed to describe retention behavior and polarity‐driven interactions. For example, linear free energy relationship models [8, 9] have focused on analyte‐stationary phase interactions, capturing how polarity influences adsorption and desorption processes. Similarly, solvent strength models [10, 11] have been widely used in reversed‐phase liquid chromatography. These models are explicitly designed for binary aqueous‐organic systems, where polarity differences between water and organic solvents are key drivers of retention behavior.

Thin‐layer chromatography (TLC) provides critical insights into the behavior of organic molecules in various organic solvent environments. Polarity is a key factor in TLC, as the interactions between solutes, solvents, and the stationary phase are largely governed by differences in polar and nonpolar characteristics. However, TLC is usually labor‐intensive and involves many repetitive trials [12, 13]. Despite its importance in determining the retardation factor ($R_{f}$), previous prediction models for structure‐retardation factors have been limited by the quantity and standardization of TLC data [14, 15]. To address this challenge, our prior work introduced an automatic high‐throughput platform for TLC analysis, providing a more extensive and standardized dataset for model training [16, 17]. This enhanced dataset not only improves the performance of predictive models, enabling more accurate and robust predictions of structure‐retardation factors in TLC experiments, but also facilitates a better analysis of the liquid–liquid interactions between solutes and solvents.

In recent years, significant progress has been made in artificial intelligence (AI)‐assisted chemical analysis, especially in the development of quantitative structure–activity relationships (QSAR) prediction models [18, 19, 20, 21, 22] and quantitative structure–property relationships (QSPR) prediction models [23, 24, 25]. While these models have achieved remarkable prediction accuracy, the AI models and feature representations they employed often lack interpretability, hindering a deeper understanding of the underlying relationships within the dataset.

The lack of transparency not only restricts the scientific impact of these models, but also undermines researchers' ability to fully trust and understand their predictions [26, 27, 28]. To address this concern, there is an increasing need for (1) transparent models and (2) interpretable feature representations that not only achieve high performance but also align with human reasoning and provide meaningful insights.

One strategy to build transparent models is to articulate explicit formulas between the $R_{f}$ values and the solute molecular structures as well as the eluent solvents in TLC analysis. Symbolic regression (SR) has recently emerged as an effective approach for unveiling complex relationships within datasets [29, 30, 31, 32, 33], and has significantly contributed to many scientific discoveries [34, 35, 36, 37, 38, 39, 40]. However, applying symbolic regression to datasets with a large number of variables remains challenging, as increasing the number of variables often leads to decreased performance and overly complex formulas, which undermines interpretability. For example, as shown in Supporting Information, when symbolic regression is directly applied to commonly used feature sets, such as physicochemical descriptors, the resulting models have limited predictive performance and reduced interpretability due to the high dimensionality.

In terms of feature representations, molecular fingerprints, such as MACCS key molecular fingerprints [41], have been often used in AI models. However, these representations are typically high‐dimensional and lack interpretability, making it difficult to understand how specific molecular structures influence the model prediction.

**FIGURE 1:**
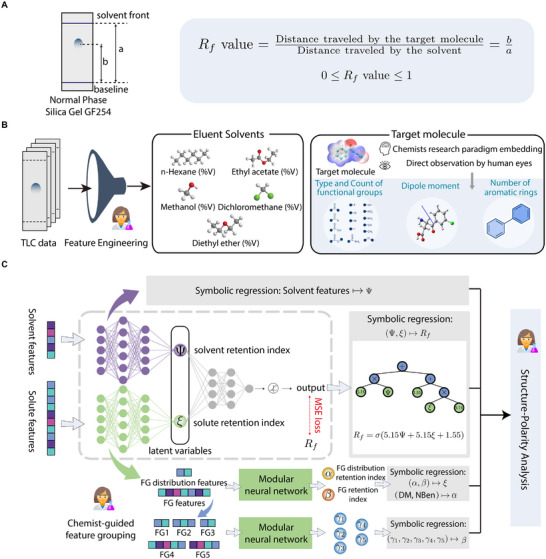
Overview of Unsupervised Hierarchical Symbolic Regression (UHSR). (A) Illustration of TLC experiment and the calculation of the retardation factor ($R_{f})$. (B) Feature engineering, involving five solvent features based on volume percentages and the decomposition of target molecules into functional groups (FGs). The molecular structure is treated as a composite formed by stacking various functional group modules. (C) UHSR framework with three main stages: chemist‐guided feature grouping, modular neural network for latent variable extraction (e.g., solute retention index ξ), and symbolic regression for discovering explicit equations between the target value and latent variables.

In this work, we present unsupervised hierarchical symbolic regression (UHSR) (see Figure 1), a new SR approach guided by modular neural networks. UHSR introduces novel retention indices, e.g., solvent retention index and solute retention index, which are learned through a modular neural network. Guided by domain knowledge, this model comprises multiple sub‐models that govern the mapping relationships between a set of input variables, e.g., solvent compositions, and a specific retention index, e.g., solvent retention index, as well as the mapping between retention indices and the output variable $R_{f}$. It is noteworthy that this process aligns with the chemists' thinking processes. The human brain struggles with high‐dimensional information, opting not to directly comprehend intricate mappings but rather to decompose and analyze through individual sub‐models. Then, we can apply the SR method on the output $R_{f}$ and the retention indices, effectively reducing the dimension of the input variables of the SR algorithm. Our experiments on the TLC benchmark dataset [17] demonstrate that UHSR achieves competitive prediction performance comparable to DNNs while providing concise formulas linking the $R_{f}$ value (indicative of the molecular polarity) with molecular structure features. Importantly, the retention indices and governing equations are evaluated by expert chemists, highlighting the alignment between the decision‐making process of UHSR and that of chemists. Additionally, we extend the scope of our study to other chemical property prediction tasks, such as spectra‐property prediction [42], where our framework also demonstrates high predictive accuracy and interpretability.

## 2 Results

### 2.1 Data‐Driven Explainable Retention Indices

It is essential for chemists to accurately characterize the overall polarity of molecules, as polarity influences molecular interaction, solubility, and chromatographic behavior. For this purpose, there are many known descriptors, such as the molecular polarity index (MPI), which is based on theoretical calculations. While effective in some cases, these descriptors have some limitations. For example, they are usually computationally expensive, especially for large or complex molecules. Also, they typically provide only global polarity information, failing to capture the localized effects of individual functional groups or their specific contributions to intermolecular interactions. Another way to estimate the molecule's polarity is based on TLC experiments. This technique directly measures the $R_{f}$ value, which reflects how a molecule interacts with both the stationary and the mobile phase under specific experimental conditions. TLC experiments are relatively simple, low‐cost, and widely accessible compared to computational methods, which often require advanced software, high‐performance computing resources, and expert knowledge. By varying the solute or solvent composition and evaluating the resulting $R_{f}$ values, chemists can systematically study polarity in a way that is directly applicable to experimental workflows.

Building on the strengths of TLC as a practical and experimentally grounded approach to studying molecular polarity, we identified several limitations in traditional methods of analyzing TLC data, which motivated this study. While TLC experiments provide valuable $R_{f}$ values that reflect molecular polarity, these raw experimental outputs are often difficult to interpret quantitatively and lack the ability to systematically separate solvent and solute contributions to retention behavior. Therefore, we developed a data‐driven method to further refine the analysis. Using the TLC dataset, we extract two retention indices, namely Ψ and ξ, acting as empirical descriptors providing information about the polarity of the solvent system and the solute molecule, respectively. The clear chemical differentiation between solutes and solvents, alongside their near‐zero Spearman correlation, compellingly validates our statistical methodology, as detailed in the Figure S1.

Importantly, compared to traditional physicochemical descriptors, our proposed indices are directly rooted in the experimental TLC dataset, which ensures that these indices are reflective of real‐world molecular interactions. Furthermore, ξ provides a localized and detailed perspective on molecular polarity, e.g., how specific functional groups impact the polarity through their specific contributions to solute‐solvent and solute‐stationary phase interactions. This detailed resolution allows ξ to capture subtle variations in polarity that are often overlooked or averaged out by traditional global descriptors.

#### 2.1.1 Chemically‐Intuitive Feature Engineering

Rather than employing molecular fingerprints or conventional physicochemical descriptors like common machine learning does, our approach deliberately labels functional groups, just like human chemists typically do. This makes our features inherently interpretable and accessible to experimental chemists. Specifically, this study encompasses an array of solute molecules, including those containing carbonyl, hydroxyl, amino, halogen, nitro, and cyano functionalities. This enables a tailored analysis of how these functional groups influence the compound's $R_{f}$ value across various solvents in terms of type, number, and allocation. For notational simplicity, we utilize the abbreviations from Table 1 for the features employed in our study. This table also provides the descriptions and statistics of these features.

**TABLE 1:** Description and statistical analysis of the features used in this study. The abbreviations, descriptions, mean values, along with the standard deviations (Std.), are presented. Additionally, Pearson's correlation coefficients (r) are computed to quantify the linear relationship between each feature and the observed $R_{f}$ value.

Abbreviations	Description	Mean(Std.)	Pearson's r
Eluent solvent			
Hex	*n*‐Hexane concentration	0.36(0.40)	−0.54
EA	Ethyl acetate concentration	0.15(0.28)	0.29
DCM	Dichloromethane concentration	0.40(0.48)	0.21
MeOH	Methanol concentration	0.014(0.025)	0.31
Et_2_O	Diethyl ether concentration	0.071(0.22)	0.13
Solute molecule			
NBen	Number of benzene ring	1.12(0.57)	0.07
DM	Dipole moment	1.34(0.74)	−0.02
${\text{Ct}}_{\text{Ph}}$	Count of phenolic hydroxyl group	0.15(0.40)	−0.15
${\text{Ct}}_{\text{OH}}$	Count of alcoholic hydroxyl group	0.04(0.21)	−0.13
${\text{Ct}}_{\text{Al}}$	Count of aldehyde group	0.19(0.40)	−0.007
${\text{Ct}}_{{\text{CO}}_{2}H}$	Count of carboxylic acid group	0.03(0.18)	−0.17
${\text{Ct}}_{{\text{RCO}}_{2}R}$	Count of ester group	0.10(0.32)	0.03
${\text{Ct}}_{R_{2}C=O}$	Count of ketone group	0.35(0.54)	−0.06
${\text{Ct}}_{\text{ROR}}$	Count of ether group	0.17(0.47)	−0.04
${\text{Ct}}_{\text{CN}}$	Count of cyano group	0.06(0.25)	−0.01
${\text{Ct}}_{{\text{NH}}_{2}}$	Count of amine group	0.08(0.28)	−0.20
${\text{Ct}}_{{\text{NO}}_{2}}$	Count of nitro group	0.06(0.25)	0.003
${\text{Ct}}_{\text{Am}}$	Count of amide group	0.02(0.14)	−0.14
${\text{Ct}}_{\text{Me}}$	Count of methyl group	0.20(0.54)	0.10
${\text{Ct}}_{F}$	Count of fluorine	0.25(0.81)	0.07
${\text{Ct}}_{\text{Cl}}$	Count of chlorine	0.11(0.39)	0.13
${\text{Ct}}_{\text{Br}}$	Count of bromine	0.15(0.39)	0.15
${\text{Ct}}_{I}$	Count of iodine	0.12(0.33)	0.22

Our method achieves a significant dimensionality reduction, requiring only 18 distinct features for describing solute molecules, compared to MACCS key molecular fingerprints (167 dimensions). While traditional physicochemical descriptors are often low‐dimensional, they typically lack the localized and explainable representation that functional group‐based features provide. Furthermore, functional groups are well‐understood chemical entities, making our results interpretable and actionable for researchers, including entry‐level chemists.

It is worth noting that an equation could be derived from various types of descriptors, and alternative descriptors beyond functional group counts, physicochemical descriptors, could also potentially yield high accuracy. However, these alternative descriptors often require specialized knowledge or computational resources, making them less accessible and practical for experimental researchers who need actionable insights. In contrast, our features are straightforward and directly relevant to experimental chemists, therefore, they bridges the gap between computational chemistry and experimental practice.

#### 2.1.2 Retention Indices

**FIGURE 2:**
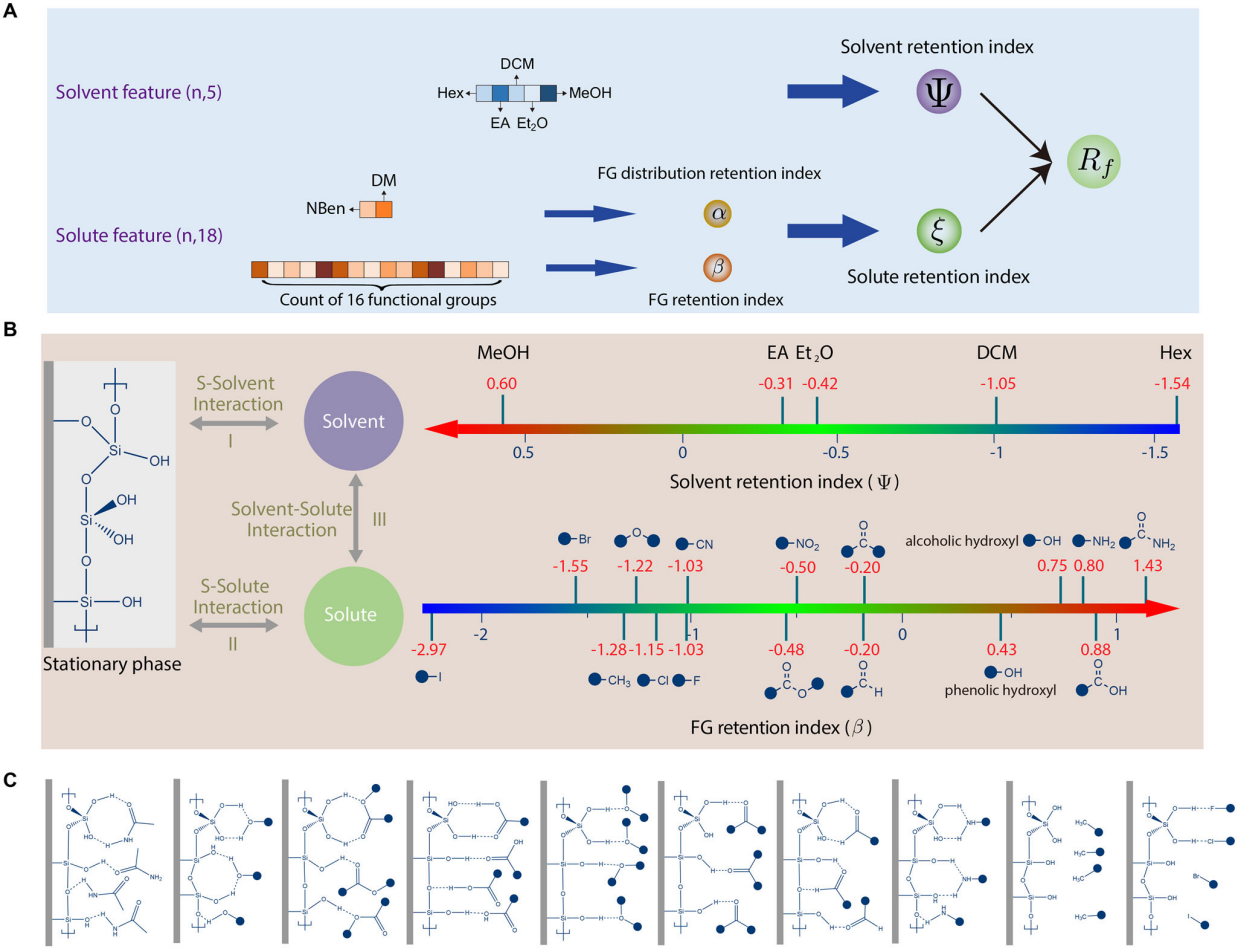
Illustration of the retention indices and their impact on chromatographic behavior. (A) The input features correspond to different retention indices. Here, FG stands for functional group. (B) TLC experiment can be understood as a process where the stationary phase (silica gel) and the mobile phase (solvent) compete for the solute molecules. This competition's outcome is reflected in the $R_{f}$ value. The factors influencing the $R_{f}$ value can be categorized into three types: interaction between the stationary phase and the solvent (I), interaction between the stationary phase and the solute (II), and interaction between the solvent and the solute (III). The two retention indices (Ψ and β) characterize how solvent and solute impact the chromatographic behavior separately. (C) Illustration of interactions between the stationary phase and different functional groups.

In this study, the stationary phase on the TLC plate is silica gel. The determined $R_{f}$ values epitomize a dynamic equilibrium. It reflects the competitive interplay between the highly polar stationary phase and the solute molecules, which are mediated through interactive forces by changing the polarities of the mobile phase during the experiment. This interplay exhibits two primary patterns: the solvent facilitates the upward transit of solute via capillary action; concurrently, solvent molecules intervene by dislodging solute molecules from the stationary phase's surface, attenuating their interactions (as illustrated in Figure 2).

The two retention indices distinctly capture the interaction dynamics between the solvent compound and the silica gel, as well as between the solute molecule and the silica gel. Importantly, our approach enables a quantitative assessment of the aforementioned interactions. In the following paragraphs, we will separately analyze these two indices.

##### 2.1.2.1 Solute Retention Index ξ

Considering that the dataset contains 387 organic compounds, previous research normally involves high‐dimensional features (such as MACCS keys with 167 dimensions) and physicochemical descriptors to encode the solute molecule into numerical values. However, the former are often challenging for human understanding, while the latter fails to meet the needs of experimental chemists for rapid polarity evaluation of new compounds. We also conducted a comparison analysis between the solute retention index ξ and several existing physicochemical descriptors, demonstrating the superiority of the solute retention index ξ in the context of TLC experiments.

To carry out this study, we carefully selected 16 representative functional groups and used their counts as features for the target molecules. Additionally, we characterized the distribution of these functional groups within the molecule by using the number of benzene rings (NBen) and dipole moment (DM). Notably, by further refining the allocation of functional groups within the benzene ring, we were able to improve prediction accuracies; the corresponding results are presented in Supporting Information.

Although we were able to reduce the feature numbers to 18, establishing an explainable connection between the polarity of the molecule and these input features remain a major challenge.

Therefore, we distinguish between two essential solute characteristics: functional group (FG) counts and their spatial distribution (Figure 2A). Both aspects are critical for elucidating molecular properties, particularly polarity, which profoundly influences molecular behavior in various solvents and reaction environments. FG counts involve 16 predefined structural motifs, offering insights into potential reactivity and solubility. While these counts provide an initial understanding of chemical behavior, molecular properties are not solely additive; linear contributions from groups alone may not fully capture the complexity of chemical phenomena. Thus, the spatial arrangement of these groups is pivotal, as it impacts the molecule's geometry, interactions, and stereochemical properties, leading to a more comprehensive characterization of molecular polarity. Therefore, we further extracted two indices, namely the FG retention index β and the FG distribution retention index α, to isolate these two effects.

Despite the polarity of functional groups being of great interest, there is a lack of quantitative and scalable methods to compare the polarities of different functional groups; in current practice, chemists often rely mainly on qualitative analysis and their experience. Our FG retention index provides the first empirical quantification of the impact of different functional groups on molecular polarity. Considering the 16 functional groups involved in this study, the input features for the sub‐model used to obtain the FG retention index β consist of 16D vectors, with each dimension representing the number of the respective functional group. To quantify the polarity of a specific functional group ${\mathrm{FG}}_{i}$, we configured the input feature vector such that the i‐th position was set to 1 and the others to 0. As illustrated in Figure 2B, the sub‐model's outputs reveal a distinct polarity order among the functional groups, i.e., amides (1.43) > carboxylic acid (0.88) > amine (0.80) > alcoholic hydroxyl (0.75) > phenolic hydroxyl (0.43) > aldehyde (−0.20) > ketone (−0.20) > ester (−0.48) > nitro (−0.50) > cyano (−1.03) > fluorine (−1.03) > chlorine (−1.15) > ether (−1.22) > methyl (−1.28) > bromine (−1.55) > iodine (−2.97).

To further validate our proposed solute retention index ξ, we conducted a Spearman correlation analysis with established molecular properties. The solute retention index exhibited a moderate positive correlation with n‐octanolwater partition coefficient (LogP, $r_{s}=0.50$), indicating that as the LogP values increase, there is a general trend of increasing the solute retention index ξ, suggesting that our index partially captures hydrophobic characteristics. Conversely, the solute retention index ξ showed a moderate negative correlation with topological polar surface area (TPSA, $r_{s}=-0.61$), highlighting that as the TPSA increases, solute retention index ξ tends to decrease, effectively capturing aspects of molecular polarity related to surface area.

For a comprehensive comparison, we employed our solute retention index ξ, MPI, LogP, TPSA, and DM as 1D solute characteristics, using five different solvent volume percentages as 5D solvent characteristics in a model established through the XGBoost model. This analysis is entirely independent of our UHSR model and was conducted to evaluate the predictive capability of ξ in a separate machine learning context. The XGBoost model served as a robust benchmark for evaluating the predictive capability of different solute characteristics.

Specifically, we compared the predictive performance of the XGBoost model, as shown in Table 2a. The solute retention index ξ demonstrated superior predictive performance, outperforming other solute descriptors. We also performed tree‐based feature importance analyses within the XGBoost model, as detailed in Figure S1. These analyses highlighted the predominant influence of the solute retention index ξ over other traditional descriptors.

**TABLE 2:** Comparison of (a) solute retention index ξ and (b) solvent retention index Ψ with established physicochemical descriptors.

(a) Comparison of model performance of solute retention index ξ with other established physicochemical descriptors				
Solute feature (with fixed 5 solvent features)	$R^{2}$	RMSE	$r_{s}$ (with $R_{f}$ value)	$r_{s}$ (with solute retention index ξ)
Solute retention index ξ	0.95	0.070	0.63	—
LogP	0.79	0.15	0.33	0.50
MPI	0.81	0.14	−0.23	−0.34
Dipole Moment	0.73	0.18	−0.06	−0.08
TPSA	0.81	0.15	−0.40	−0.61

(b) Comparison of solvent retention index Ψ with estabilished solvent properties					
Solvent	HBD	HBA	Solubility in water (g/100g) [43]	LogP	Ψ
Hex	0	0	0.01	3.42	−1.54
DCM	0	0	1.32	1.01	−1.05
${\mathrm{Et}}_{2}O$	0	1	6.9	0.76	−0.42
EA	0	2	7.7	0.29	−0.31
MeOH	1	1	Miscible	−0.27	0.60

##### 2.1.2.2 Solvent Retention Index Ψ

Chromatographic retention on silica gel results from the synergistic interplay of electrostatic (dipole–dipole and ion–dipole), hydrogen‐bonding, and van der Waals dispersion forces; molecular polarity alone represents only one component of this adsorption mechanism. Accordingly, we define the solvent retention index Ψ as an empirical measure of the net adsorption strength of each pure solvent on silica.

Methanol (MeOH), with one hydrogen‐bond donor (HBD) and one acceptor (HBA), forms both six‐ and eight‐membered chelates with the surface silanol and siloxane groups (see Figure 2C). Ethyl acetate (EA) features two HBAs that engage in an eight‐membered ring complex via its ester oxygens. Diethyl ether (${\mathrm{Et}}_{2}O$) has a single HBA, allowing only one hydrogen‐bonding mode. Dichloromethane (DCM), bearing two chlorides, interacts primarily through weaker dipole–dipole and dispersion forces. In contrast, *n*‐hexane (Hex) adsorbs only via weak dispersion interactions and cannot displace more strongly adsorbing solvents. Under this framework, the adsorption hierarchy is

$$\mathrm{MeOH}>\mathrm{EA}>{\mathrm{Et}}_{2}O>\mathrm{DCM}>\mathrm{Hex}.$$

The input features of the sub‐model used to obtain Ψ consist of 5D vectors, with each dimension representing the volume percentage of a solvent compound. Consequently, variations in the compound ratio within the solvent system directly influence the value of Ψ, thereby changing the polarity of the solvent. Moreover, to isolate the effect of each compound, we can assign the value 1 to the volume percentage that corresponds to the target solvent compound, while setting the others to 0. This approach yields the following values (see Figure 2B):

$$\begin{array}{cc} & \Psi_{\mathrm{Hex}}=-1.54,\;\Psi_{\mathrm{DCM}}=-1.05,\;\Psi_{{\mathrm{Et}}_{2}O}=-0.42,\\ & \Psi_{\mathrm{EA}}=-0.31,\;\Psi_{\mathrm{MeOH}}=0.60 \end{array}$$

Comparison with experimental miscibility indices (LogP and aqueous solubility [43]) confirms that Ψ captures the combined electrostatic, hydrogen‐bonding, and dispersion contributions to solvent–silica adsorption (see Table 2b).

### 2.2 The $R_{f}$ Governing Equation

**FIGURE 3:**
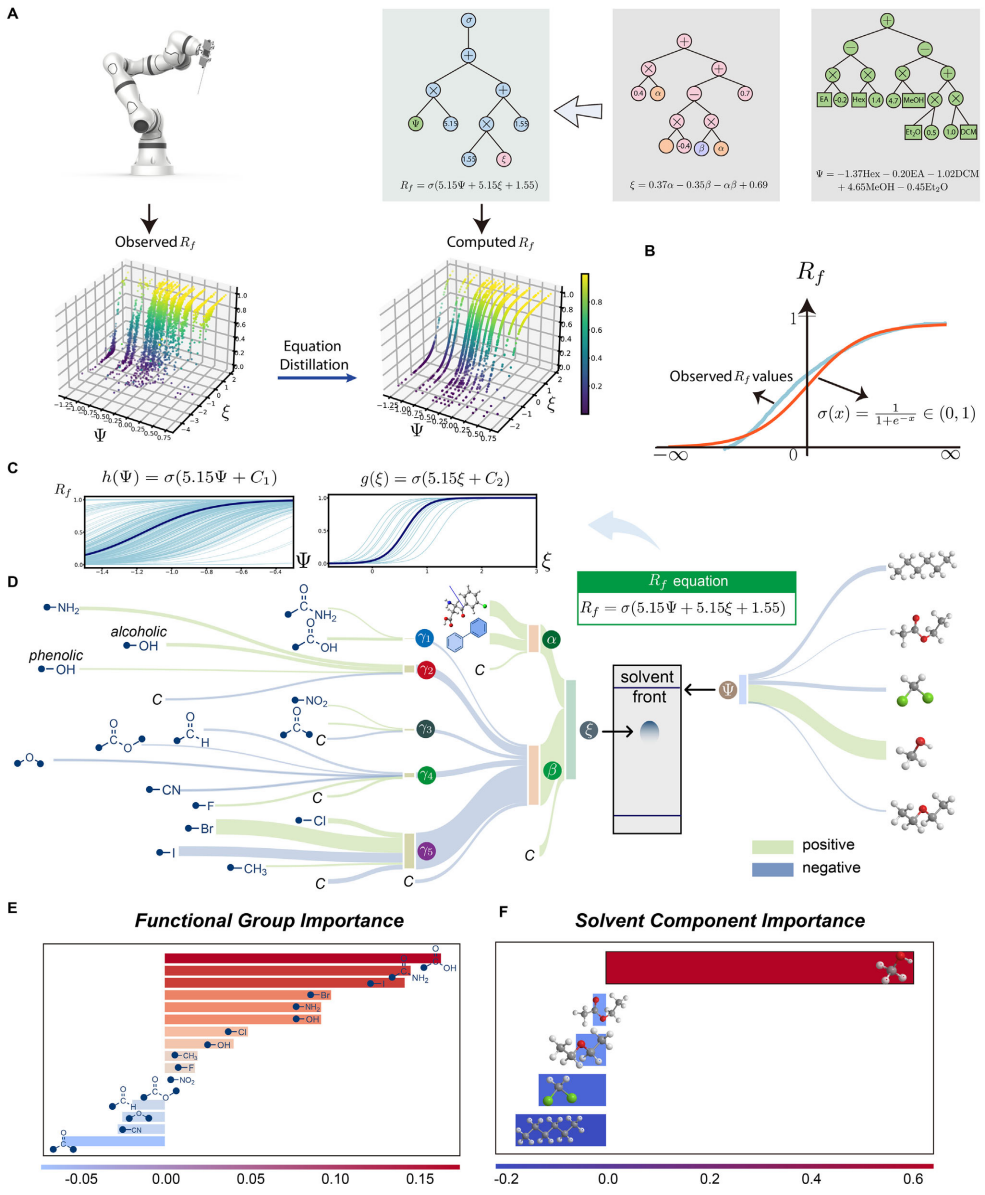
Visualization of the latent variables and the decomposition of the retrieved formula. (A) Visualization of the observed and calculated $R_{f}$ values with two retention indices solvent retention index Ψ and solute retention index ξ. (B) Fitting observed $R_{f}$ values with a Sigmoid function. (C) Decomposition of Equation 1 into h(Ψ) and g(ξ). (D) Visualization the flow of how input variables contribute to the $R_{f}$ value, here C represents a constant. (E) Importance of functional groups in determining the functional group retention index (β). (F) Importance of solvent components in determining the solvent retention index (Ψ).

To comprehensively understand the relationship between the $R_{f}$ value and the two retention indices, we used symbolic regression to formulate empirical mathematical expressions connecting the $R_{f}$ value and the two retention indices (Figure 3A). The observed $R_{f}$ values were obtained directly from our automatic high‐throughput platform [17], while the computed $R_{f}$ values were calculated from the $R_{f}$ governing equation (see Equation 1 below).

To ensure that the computed $R_{f}$ values lie within the range from 0 to 1, a sigmoidal function was employed (see Figure 3B). By using symbolic regression, one obtains several candidates for the $R_{f}$ governing equation (see Table S1). Since a simple formula is desired, and considering the prescribed fitting accuracy ($R^{2}=0.908$, RMSE =0.104), we selected

(1) $$R_{f}=\sigma \left(5.15\Psi +5.15\xi +1.55\right),\;\sigma \left(x\right)=\frac{1}{1+e^{-x}}.$$

In order to separately analyze the individual influence of solvent and solute on the $R_{f}$ value, we further decomposed the formula for $R_{f}$ in Equation 1 into h(Ψ) and g(ξ). For instance, as illustrated in Figure 3C, when considering a specific solute molecule (i.e., fixing a value for the solute retention index ξ), we can reformulate Equation 1 as $R_{f}=h\left(\Psi \right)=\sigma \left(5.15\Psi +C_{1}\right)$, where $C_{1}$ depends on the chosen solute molecule. The pattern observed in Figure 3C shows that $R_{f}$ increases with the solvent retention index Ψ. Since a larger value of solvent retention index Ψ indicates greater polarity, this finding emphasizes that the solvent retention index Ψ correlates with the polarity of the solvent. Furthermore, we observe that the shape of h(Ψ)’s graph varies significantly for different solute molecules. In particular, Figure 3C indicates that, within the context of our dataset, changing the solvent's composition only has a subtle effect on $R_{f}$ for solute molecules with extreme polarities. Similarly, when the solvent is fixed, Equation 1 can be reformulated as $R_{f}=g\left(\xi \right)=\sigma \left(5.15\xi +C_{2}\right)$, where $C_{2}$ depends on the chosen value for the solvent retention index Ψ. By analysis similar to the previous case (fixed solute), the $R_{f}$ value increases with the solute retention index ξ. But now, a larger value of the solute retention index ξ indicates a lower polarity. Moreover, we observe that the shape of g(ξ)’s graph is of the same *S*‐shaped type across different solvent systems (see Figure 3C).

Due to the limitations of the data, different modular neural networks can achieve similar accuracy. However, we observed that while the retention indices change, the discovered $R_{f}$ equations maintain a similar form with varying parameters, as illustrated in Tables S10–S12. In TLC experiments, the absolute values of the retention indices are less crucial than their relative values. The relative values of the retention indices are what guide the separation process in TLC. Given that the underlying equations maintain a similar form, variations in the retention indices can be viewed as differences in units rather than changes in fundamental properties. For example, given a target molecule and two solvent systems (e.g., the polarity of the first solvent system is smaller than the second solvent system), we may obtain two sets of solvent retention indices $\left(\Psi_{1},\Psi_{2}\right)$ and $\left(\Psi_{1}^{\prime },\Psi_{2}^{\prime }\right)$, the relative relationship maintain the same, i.e., $\Psi_{1}<\Psi_{2}$, $\Psi_{1}^{\prime }<\Psi_{2}^{\prime }$.

### 2.3 Quantification of Solvent Polarity

In addition to characterizing the polarity of the eluent solvent, the UHSR framework offers an explainable solution for understanding how different compounds influence the solvent retention index Ψ. In this study, we consider five distinct solvent compounds: Hex, EA, DCM, MeOH, and ${\mathrm{Et}}_{2}O$. To achieve the designed solvent polarity, chemists usually adjust the ratios of these compounds in the mobile phase system. By directly applying the SR algorithm, we obtained the empirical mathematical equation between solvent retention index Ψ and its corresponding input variables, i.e., the volume ratio of Hex, DCM, ${\mathrm{Et}}_{2}O$, EA, and MeOH in the mobile phase system. The following formula achieved a high $R^{2}$ of 0.956 and a low RMSE of 0.069 with a simple form,

(2) $$\Psi =-1.37\mathrm{Hex}-0.20\mathrm{EA}-1.02\mathrm{DCM}+4.65\mathrm{MeOH}-0.45{\mathrm{Et}}_{2}O.$$

Specifically, there are three mobile phase systems involved in the dataset, which are Hex/EA, $\mathrm{Hex}/{\mathrm{Et}}_{2}O$, and MeOH/DCM. Equation 2 quantifies the impact of the two compounds on solvent polarity within each system. For instance, in the presence of Hex and EA, the solvent retention index Ψ is calculated as Ψ=−1.37Hex−0.20EA. As the volume of EA increases and the volume of Hex decreases, the solvent polarity exhibits a corresponding increase. Moreover, the constant term of 4.65 with MeOH indicates that even a slight increase/decrease in the volume of MeOH will lead to a significant increase/decrease in solvent polarity.

### 2.4 Quantification of Solute Polarity

As discussed earlier, we introduce two additional functional group‐related indices, FG distribution retention index α and FG retention index β, to enhance our understanding of the relationship between the molecular polarity and the molecular structure. With the help of symbolic regression, we obtained an explicit mathematical relationship between ξ and (α,β) as follows:

(3) ξ=0.37α−0.35β−αβ+0.69.

Breaking down Equation 3, the molecular polarity can be understood as a combination of the effect of functional groups' distribution ($f_{1}\left(\alpha \right)=0.37\alpha$), the contribution of the functional groups within the molecule ($f_{2}\left(\beta \right)=-0.35\beta$) and the interaction between these two factors ($f_{3}\left(\alpha ,\beta \right)=-\alpha \beta$).

To perform a finer‐grain analysis of the individual impacts of α and β on molecular polarity, we further derived specific equations that decompose the contributions of each feature, as follows,

(4a) $$\begin{array}{cc} \alpha & =\left(\mathrm{DM}+\mathrm{NBen}-0.29\right)(-\mathrm{NBen}\cdot e^{\mathrm{DM}-\mathrm{NBen}}+2.28\cdot e^{\mathrm{DM}-\mathrm{NBen}}\\ & \;-0.64\mathrm{NBen}+1.66)-0.74, \end{array}$$

(4b) $$\begin{array}{cc} \beta & =(0.07\gamma_{2}-\left(0.003\gamma_{5}\left(2\gamma_{2}-\gamma_{3}-\gamma_{4}\right)-0.27\right)\\ & \;\cdot (\gamma_{1}-\gamma_{5}+\left(\gamma_{2}-\gamma_{4}\right)\left(0.14\gamma_{5}+0.35\right)))-0.63, \end{array}$$

where

(5) $$\left\{\begin{array}{cc} \gamma_{1} & =5.16{\text{Ct}}_{\text{Am}}+5.16\log\left({\text{Ct}}_{{\text{CO}}_{2}H}+0.86\right),\\ \gamma_{2} & =3.83{\text{Ct}}_{\text{Ph}}+8.70\left({\text{Ct}}_{{\text{RNH}}_{2}}+{\text{Ct}}_{\text{OH}}\right)-0.73,\\ \gamma_{3} & =\frac{{\text{Ct}}_{{\text{NO}}_{2}}+{\text{Ct}}_{{\text{RCO}}_{2}R}}{0.44{\text{Ct}}_{{\text{RCO}}_{2}R}-0.14}-0.29,\\ \gamma_{4} & ={\text{Ct}}_{F}\cdot \left({\text{Ct}}_{\text{ROR}}+1.44\right)-2.96{\text{Ct}}_{R_{2}C=O}-2.96e^{{\text{Ct}}_{\text{al}}}\\ & \;-2.96\left({\text{Ct}}_{\text{al}}+0.66\right)\cdot \left({\text{Ct}}_{R_{2}C=O}+{\text{Ct}}_{\text{ROR}}+{\text{Ct}}_{\text{CN}}-1.20\right),\\ \gamma_{5} & ={\text{Ct}}_{\text{me}}+2.54{\text{Ct}}_{\text{Cl}}+2.54\left(1.21-{\text{Ct}}_{\text{Br}}\right)\\ & \;\times ({\text{Ct}}_{\text{Br}}+2.73\left({\text{Ct}}_{I}-0.52\right))+2. \end{array}\right.$$

The above equations are indeed more complex and less directly interpretable than the top‐level $R_{f}$ equation (Equation 1). This complexity arises from the intricate nature of molecular polarity, which involves nonlinear interactions between functional groups and structural features. While the top‐level $R_{f}$ equation is designed to provide a concise, human‐readable relationship between solute polarity, solvent polarity, and the $R_{f}$ value, serving as a conceptual tool for understanding how polarity indices influence $R_{f}$ values, the more detailed Equations (4) and (5) serve a different purpose. These equations function as computational tools to calculate latent chemical properties, such as α and β, from molecular structures. By incorporating terms like $\gamma_{1}$ to $\gamma_{5}$, they enable precise quantification of the contributions of specific functional groups and their interactions, which would otherwise be difficult to disentangle.

Although these detailed equations are not intended for direct human interpretation, their value lies in supporting downstream analyses, such as feature importance assessments and visualizations. These analyses, introduced in the following subsection, help bridge the gap between computational complexity and practical insights, making the results more accessible and actionable for researchers.

### 2.5 Visualization of the Discovered Equations

To understand how the discovered equations explain the relationship between input features and the final $R_{f}$ value prediction, we utilized two complementary visualization approaches: feature importance visualization and direct value contribution visualization. These methods provide different but synergistic perspectives on how features influence the final $R_{f}$ values.

Feature importance quantifies the sensitivity of the model output to variations in individual input features. Through the derived equations, we can directly identify which features have the most significant impact on the $R_{f}$ prediction. For example, as shown in Figure 3E, the count of halogen atoms (e.g., iodine) and the carboxylic acid group (${\mathrm{Ct}}_{{\text{CO}}_{2}H}$) are identified as features with high importance. As shown in Figure 3F, methanol (MeOH) plays a significant role in influencing the $R_{f}$ values. This approach is particularly useful for identifying the key drivers of variability in the model's predictions across the dataset.

In addition to understanding feature importance, we also visualized the direct numerical contribution of each input variable to a specific prediction. This approach traces how the “value” of each feature propagates through the discovered equations to influence the final $R_{f}$ value. As shown in Figure 3D, this step‐by‐step visualization reveals how input variables contribute to intermediate indices (e.g., $\gamma_{1}$, β, ξ) and ultimately to $R_{f}$.

For instance, if the count of iodine is set to 1, we can directly observe how this value influences $\gamma_{5}$, which then propagates to β, and finally contributes to the $R_{f}$ value. Unlike feature importance, which focuses on sensitivity to changes, this approach provides a numerical breakdown of how much each input feature contributes to the final prediction for a given input configuration.

While feature importance highlights which features are most critical across the dataset, direct value contribution visualization, provides a more detailed view of how the equations process specific inputs. Together, these visualizations offer a comprehensive understanding of the discovered equations, allowing us to both identify key features and trace their influence on the $R_{f}$ value.

### 2.6 Chemists' Evaluation on UHSR

#### 2.6.1 Survey Evaluation

To thoroughly assess whether the UHSR model thinks like chemists, we grounded our evaluation using the judgments of chemists. We conducted a comprehensive survey involving 100 chemists with an average of 10 years of experience in the field of chemistry. Please see the complete survey in the Supporting Information.

**FIGURE 4:**
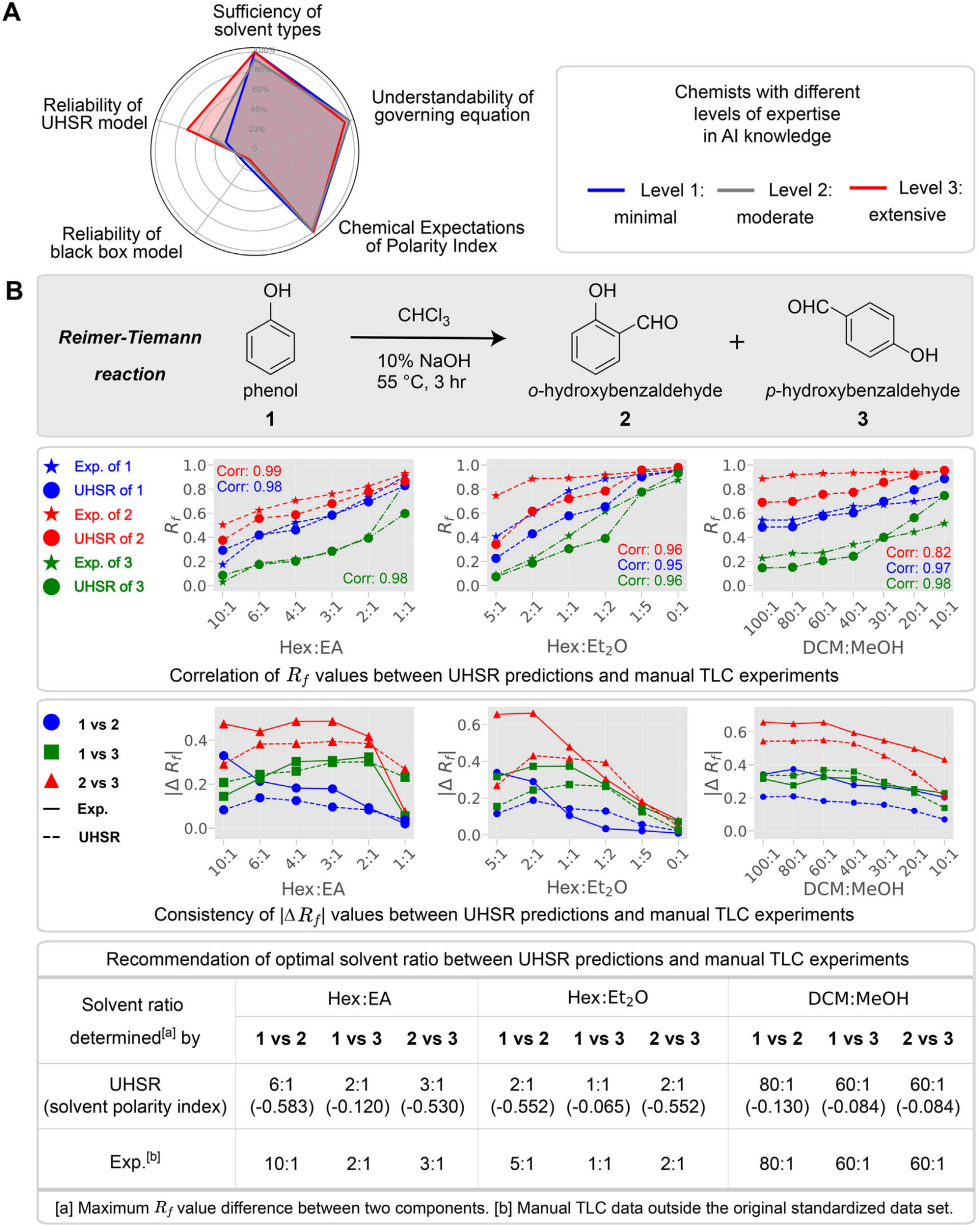
Analysis of the survey and model‐experimental data correlation. (A) Survey analysis from chemists with varying levels of AI expertise. (B) Assessment of the consistency between the UHSR model (AI model trained on standardized data) and manual experimental (Exp.) data (non‐standardized experimental conditions, such as different TLC plates, temperatures, humidity levels, and time) on the optimal solvent ratio.

According to the survey results, an impressive 97.0% of the chemists indicated that the retention indices derived from the UHSR model align well with their expectations based on their professional experience (see Figure 4A). This high level of alignment shows the model's ability to capture key aspects of molecular polarity that are consistent with established chemical principles. Similarly, 99.0% of the chemists agreed that the $R_{f}$ governing equation meets their expectations. This suggests that the model's predictions are not only accurate but also resonate with the intuitive understanding of chemists. 99.0% of the respondents felt that the retention indices and the governing equation provided new insights and knowledge. These results indicate that the qualitative concepts chemists had accumulated over time align with the quantitative equations we discovered. Our quantitative equations provide a structured and solidified description of the intuitive conclusions that chemists have developed through their experiences. This solidification of qualitative concepts into quantitative forms offers a valuable bridge between empirical knowledge and rigorous scientific modeling.

To better understand chemists' preference between the UHSR model and traditional machine learning models (such as artificial neural networks), we categorized the chemists into three levels based on their levels of expertise in AI knowledge [44]: Level 1 indicates minimal AI knowledge, Level 2 indicates moderate AI knowledge, and Level 3 indicates extensive AI knowledge. As shown in Figure 4A, among chemists with Level 3 AI knowledge, 88.2% of chemists expressed a higher level of trust in the UHSR model compared to traditional machine learning models, and the remaining showed no preference between the two types of models. Among chemists with Level 2 AI knowledge, 44.6% trusted the UHSR model more, 43.1% had no preference, and only 12.3% trusted the black box model more. For chemists with minimal AI knowledge, 55.6% of the chemists showed no preference, while 22.2% trusted the UHSR model more. This finding suggests that the transparency and interpretability of the UHSR model are particularly appreciated by those with a deeper understanding of AI.

#### 2.6.2 Experimental Evaluation

Furthermore, it is crucial to recommend solvent ratios for column chromatography by examining the positioning of compounds in TLC experiments under various solvent ratios. Ideally, the solvent ratio should maximize the difference in $R_{f}$ values between the components to be separated, or place the target molecule's $R_{f}$ value between 0.2 and 0.3 [45]. Based on this approach, we explored the consistency of the optimal solvent ratio between the UHSR model (an AI model trained from standardized data) and experimental data under non‐standardized experimental conditions (such as different TLC plates, temperature, humidity, and time). Here, we examined the product *o*‐hydroxybenzaldehyde **2** and *p*‐hydroxybenzaldehyde **3** of the Reimer–Tiemann reaction [46] from phenol **1** were detected by thin‐layer chromatography (Figure 4B). At different solvent ratios, there was a high correlation between $R_{f}$ values of the manual TLC experiment (Exp.) and predicted values of UHSR. By analyzing the $R_{f}$ differences between component pairs across 19 different solvent ratios for five solvents, we found that 7 out of 9 optimal solvent ratios recommended by UHSR predictions matched the experimental data, demonstrating the robustness of our model under a variety of experimental conditions. Additionally, the quantification of solvent polarity for the recommended optimal solvent ratios was determined using the proposed solvent retention index Ψ. It was observed that some of these optimal solvent ratios exhibit similar polarity.

### 2.7 Transferability and Broader Applications of the UHSR Framework

#### 2.7.1 Extension to Column Chromatography

Column chromatography (CC) can be viewed as a natural preparative‐scale extension of the structure–polarity principles that govern TLC behavior. To evaluate the effectiveness of our UHSR framework in CC, we employ the same chemically interpretable molecular features listed in Table 1 (e.g. functional group counts) alongside solvent composition parameters (volume percentages of mobile phase components), utilizing experimental data from an automated CC platform [47].

In CC optimization, two critical volume metrics are defined: $v_{1}$, the eluent volume consumed when the target compound begins eluting (corresponding to retention factor k), and $v_{2}$, the total eluent volume required for complete compound elution (incorporating peak broadening effects). These volumes are predicted through polarity‐governing equations:

$$v_{1}=0.25\theta {\left(\frac{\theta }{\mu }\right)}^{3}-0.27\theta \frac{\theta }{\mu }+0.27\theta +6.50,$$

$$\begin{array}{cc} v_{2} & =0.006{\left(\frac{\theta \left(\theta +\mu \right)}{\mu -1}-13.9\right)}^{2}+0.003{\left(\theta +0.86\mu +1.72\right)}^{2}\\ & \;+1.94\frac{\mu }{\theta }-25.5\frac{1}{\theta }+25.1, \end{array}$$

where θ represents the learned solvent polarity index (mobile phase) and μ represents the learned solute polarity index (analyte).

The equations exhibit competitive predictive performance compared to “black‐box” models, such as artificial neural networks (ANN), as reported in [47]. Specifically, $v_{1}$ achieves an $R^{2}=0.841$ and $v_{2}$ achieves an $R^{2}=0.834$.

#### 2.7.2 Beyond Polarity Anlysis

Although in this work, we focused on $R_{f}$ prediction in TLC as a case study, the UHSR framework is highly transferable and can be applied to a wide range of property prediction tasks beyond polarity analysis. To demonstrate this, we extended the framework to spectra‐property prediction [42].

The spectra‐property dataset focused on predicting thDCMe adsorption energy ($\Delta E_{ads}$) and charge transfer (Δe) of CO‐adsorbed Cu‐based MOF systems from their infrared and Raman spectra. Specifically, we selected CO@CuBTC dataset, where each sample consists 18 features, including six frequency values ($f_{1},\cdots ,f_{6}$), their corresponding IR values ($I_{1},\cdots ,I_{6}$), and Raman values ($R_{1},\cdots ,R_{6}$).

Using the modular neural network within the UHSR framework, these features were categorized into three indices: frequency index ($\hat{f}$), IR index ($\hat{I}$), and Raman index ($\hat{R}$). The resulting governing equation for adsorption energy ($\Delta E_{ads}$) and charge transfer (Δe) are as follows,

$$\begin{array}{cc} \Delta E_{\mathrm{ads}} & =\frac{-2\hat{f}+8.75\hat{I}-18.5\hat{R}}{{\hat{f}}^{2}},\\ \Delta e & =-0.00993\hat{f}-0.00165{\hat{R}}^{2}-0.00993\hat{R}-\frac{0.0243}{\hat{I}}. \end{array}$$

These equations achieve impressive performance, with $R^{2}=0.862$, RMSE=0.045 for adsorption energy, and $R^{2}=0.990$, RMSE=0.005 for charge transfer, respectively. Notably, these results surpass the baseline performance reported in [42], where $R^{2}=0.834$, RMSE=0.055, and $R^{2}=0.938$, RMSE=0.013.

This case study demonstrates the ability of UHSR to adapt seamlessly to different tasks with high performance and interpretability.

## 3 Methods

### 3.1 TLC Dataset

Several variables contribute to the variation in $R_{f}$ values, including factors such as the nature of the stationary phase, temperature, and humidity. Traditional methods of obtaining TLC data manually suffer from a lack of standardization, making scalability challenging. To address this issue, our prior work [17] introduced a robotic platform designed for high‐throughput collection of TLC data (Figure 3A), which not only ensured efficiency in data acquisition but also established a standardized and scalable methodology.

The TLC dataset comprised in total 4944 measurements of the $R_{f}$ values, involving 387 organic compounds and three mobile phase systems. These mobile phase systems were *n*‐Hexane/Ethyl acetate, Diethyl ether/*n*‐Hexane, and Methanol/Dichloromethane systems, with a total of 17 different solvent compositions.

### 3.2 Framework of UHSR

**FIGURE 5:**
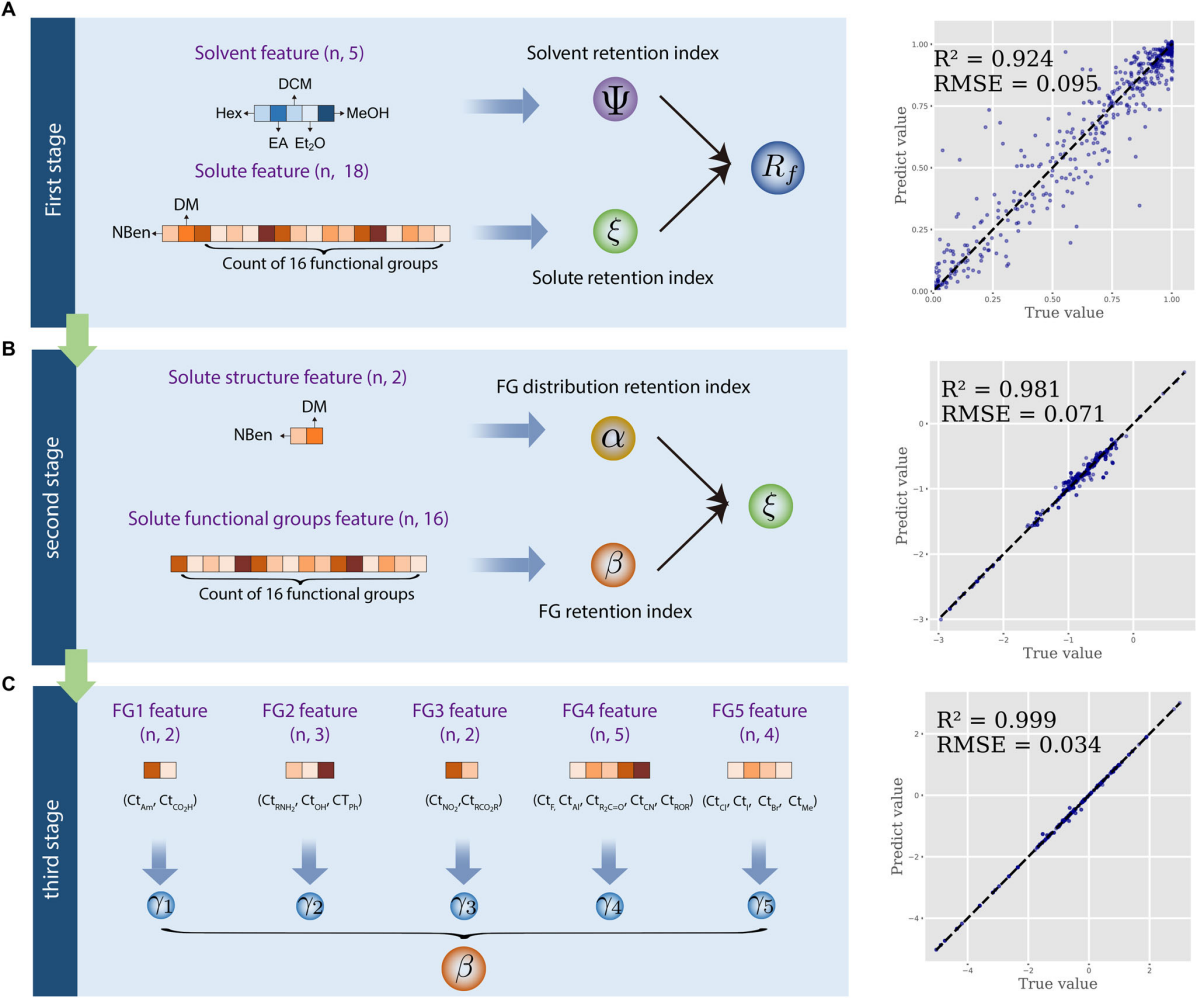
Hierarchical structure of learning latent variables. Three stages are organized from top to bottom. (A) At the first stage, two latent variables, Ψ and ξ, are generated to encapsulate the overall polarity of the solvent and the solute, respectively. (B) At the second stage, two additional latent variables, α and β, are learned to assess the solute molecule's polarity from two distinct perspectives. α is linked to the distribution of functional groups, while β pertains to the quantity of individual functional groups. (C) The third stage characterizes five latent variables, each representing the impact of specific groups of functional groups.

To address the inherent complexity imbalance between solvent and solute compounds, our framework, unsupervised hierarchical symbolic regression (UHSR), was strategically organized hierarchically in a top‐to‐down manner. It was motivated by the rationale grounded in established practices observed in TLC experiments. As illustrated in Figure 5, each stage focused on specific aspects of the feature space, allowing the model to learn different latent variables, which discern patterns at different levels of granularity.

For instance, the top stage provided a broad overview of the interaction between solvent and solute, capturing their global polarity characteristics. Subsequent stages then delved into more detailed and specific characteristics of the solute molecular structure, offering intricate insights regarding the contributions of different molecular features to the overall molecular polarity. As illustrated in Figure 1C, the framework contained three key parts: feature engineering, modular neural network and symbolic regressions. In this subsection, we briefly introduce the algorithms and methods, the detailed information is in the Supporting Information.

#### 3.2.1 Feature Engineering

Organic molecules are composed of various functional groups arranged along their molecular skeletons. The arrangement of these groups influences the polarity of a given molecule. Organic chemists typically assess the polarity of target molecules by considering the type, number, and distribution of functional groups. Here, we focused on 16 representative functional groups (Figure 1C). Besides the type and number of functional groups, their spatial distribution is also crucial for molecular polarity. To account for this, we included additional descriptors such as the number of aromatic rings and dipole moments. The reasons for choosing the other three descriptors are as follows:

- **DM (Dipole Moment)**: The dipole moment is a critical descriptor as it reflects the overall polarity of a molecule, indicating how electron density is distributed. However, the dipole moment alone might not fully capture the complexity of molecular interactions, especially in varied solvent environments. Its exclusion does affect the model, but not to the extent that it could singularly determine all polarity‐related interactions. We found that combining the dipole moment with other descriptors provided a more robust prediction.
- **NBen (Number of Benzene Ring)**: The presence and number of aromatic rings significantly influence molecular properties and interactions, particularly in aromatic–aromatic stacking and van der Waals interactions. These factors are crucial in many solute‐solvent interactions that the dipole moment alone may not account for.

In terms of features related to the solvent, we considered the volume ratios (V%) of five different solvent compounds, i.e., *n*‐hexane, ethyl acetate, dichloromethane, diethyl ether, and methanol.

We conducted an input feature control experiment to evaluate the representation power of various feature sets. Two baseline feature sets were compared: one‐hot encoded categorical features and random features. One‐hot encoded features represent input features as binary vectors, where each postion indicates the presence or absence of a specific molecule. Random features, on the other hand, are randomly generated numerical values assigned to each molecule, serving as a non‐informative baseline. In addition, we tested another two types of features commonly used in statistical models: 167D MACCS keys and conventional physicochemical descriptors. Specifically, these descriptors include molecular weight (MW), topological polar surface area (TPSA), the logarithm of n‐octanol–water partition coefficient (LogP), hydrogen bond donor number (HBD), hydrogen bond acceptor number (HBA), molecular polarity index (MPI), polar surface area percentage (PSA) and dipole moment (DM).

**FIGURE 6:**
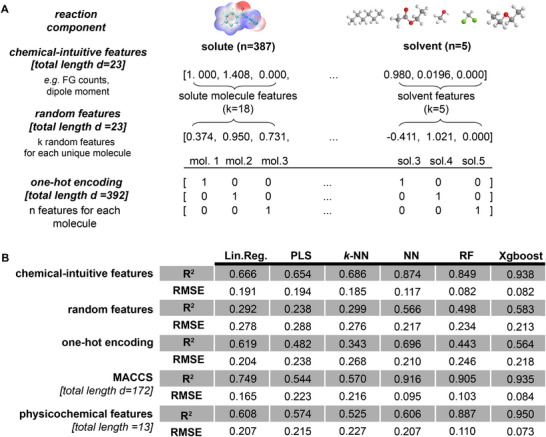
Comparison of input feature control experiments. (A) Schematic of our features vs. random features and one‐hot encoded categorical features. (B) Comparison of coefficient of determination ($R^{2}$) and RMSE values between input features.

The five sets of input features were tested on six different machine learning models, including linear regression (Lin. Reg.), partial least squares (PLS), k‐nearest neighbors (*k*NN), a simple neural network (NN), random forest (RF) and XGBoost. The results are presented in Figure 6.

#### 3.2.2 Architecture Design of Modular Neural Network

The architecture of our network was motivated by the rationale grounded in established practices observed in TLC experiments. TLC experiments conventionally entailed the execution of controlled experiments designed to meticulously dissect the influence of solute polarity and solvent polarity on $R_{f}$. To meet the specific demands of this task, we modified conventional Multi‐Layer Perceptron (MLP) architecture into a modular neural network, designed to enhance interpretability and align with domain knowledge.

*Feature Grouping*. Let us consider a specific modular neural network. Given an input sample $x\in R^{d}$, the initial step involved dividing the input features into k feature groups, denoted as $x_{1},\cdots ,x_{k}$. Each feature group $x_{i}\in R^{d_{i}}$ was non‐overlapping, and their combined dimensionality satisfied $\sum_{i=1}^{k}d_{i}=d$. These feature groups were determined using a chemist‐guided strategy, and the specific grouping applied at each stage is illustrated in Figure 5. Specifically, in the first stage, we categorized features into two distinct groups: solvent features ($x_{\text{solvent}}\in R^{5}$) and solute features ($x_{\text{solute}}\in R^{18}$). In the second stage, the solute features were further categorized into two groups: FG features ($x_{\text{FG}}\in R^{16}$) and FG distribution features ($x_{\text{FGdist}}\in R^{2}$). In the third stage, the FG features were further categorized into five distinct groups: $x_{\text{FG1}}\in R^{2}$, $x_{\text{FG2}}\in R^{3}$, $x_{\text{FG3}}\in R^{2}$, $x_{\text{FG4}}\in R^{5}$ and $x_{\text{FG5}}\in R^{4}$.

*Latent Representation and Modularity*. A key innovation of our architecture lay in the formulation of the target latent representation layer j, where $z_{i}^{\left(j\right)}=g_{i}\left(x_{i}\right)$. Each function $g_{i}$ operated independently within the modular neural network, meaning there was no parameter sharing across $g_{1},\cdots ,g_{k}$. Thus, we could consider $g_{i}$ as a sub‐model. This design ensured the preservation of feature‐specific information. The final output, $o=h(z^{\left(j\right)})$, encapsulated the information from the target latent representation layer.

This design restricted the receptive field of the latent variables, therefore ensuring that each latent variable depended solely on a specific group of input features. For instance, in the first stage, the latent variables learned from solvent features and solute features were interpreted as the solvent retention index (Ψ) and the solute retention index (ξ), respectively. These indices captured distinct contributions from the input features, reflecting their individual effects on $R_{f}$.

*Training Procedure*. The training procedure consisted of three stages, with each stage involving a modular neural network to extract retention indices that reflected different levels of granularity, as illustrated in Figure 5. In the first stage, the input features were divided into two feature groups: solvent features and solute features. These were processed independently by the modular neural network, resulting in two latent variables: the solvent retention index (Ψ) and the solute retention index (ξ). These indices represented the overall contributions of solvent and solute features to the $R_{f}$ value.

In the second stage, we focused on solute features only. The solute features were further divided into two subgroups: functional group (FG) counts and the distribution of functional groups within the molecule. The modular neural network processed these subgroups to extract two additional indices: the FG retention index (β), which captured the contribution of different FGs to $R_{f}$, and the FG distribution retention index (α), which represented the influence of how functional groups were distributed across the molecule. These indices provided a more detailed understanding of how functional groups impact molecular polarity and retention behavior.

The third stage involved a fine‐grained analysis of functional group contributions. Here, the FG features were further subdivided into five specific FG categories. Using the modular neural network, five fine‐grained FG retention indices ($\gamma_{1},\cdots ,\gamma_{5}$) were extracted. These FG retention indices captured the unique contributions of specific types of functional groups to $R_{f}$.

This hierarchical decomposition, progressing from broad solvent and solute features to detailed FG‐specific contributions, ensured that the model disentangles complex relationships between molecular features and the $R_{f}$ value.

#### 3.2.3 Symbolic Regression

SR aims to identify mathematical expressions that capture the inherent relationships within a given dataset. Unlike approaches that involve fitting parameters to overly complex general models (such as machine learning models), SR explores a space of simple analytic expressions. The goal is to discover accurate and explainable models that directly represent the underlying patterns in the data. SR is usually implemented by evolutionary algorithms, such as genetic programming (GP) [29, 30, 48].

The most common way to visualize a symbolic expression is a tree‐structure with nodes and branches, as shown in Figure 3A. This structure includes primitive functions (e.g., +,−,×,÷,exp) and terminal nodes (input features and numeric constants). Through a series of mutation operations, the GP algorithm seeks to determine the optimal number of nodes and terminals that provide the best fit to a given dataset. For our implementation of the widely used GP‐SR algorithm, we employed the open‐source Python library PySR^1^ [49]. The hyperparameter configurations for our implementation are detailed in the Tables S8 and S9. Moreover, when deriving the $R_{f}$ governing equations, we imposed a constraint requiring all parameters to be positive. If any parameter turned out to be negative, we added a minus sign before the learned retention index to standardize the results.

It is worth mentioning that our method is not limited to GP algorithms. In fact, the UHSR framework is designed to accommodate various symbolic regression methods; comparable results obtained from deep reinforcement learning based SR methods (such as DISCOVER^2^ [50]) are included in the Table S5.

### 3.3 Expert Chemist Evaluation

The expert chemist evaluation consists of a quantitative survey assessment in which chemists were provided with a QR code or website link to complete the survey (please see Supporting Information for the complete survey). The study was submitted for ethical review and received approval from the Peking University Institutional Review Board (No. IRB00001052‐2408). Participation was not incentivized with compensation, as it was considered to contribute to the general advancement of research. The study was anonymous, and only the lead researcher had access to the assignment of the experimental IDs. Participants were chemical professionals, including faculty members, industry chemists, and graduate students, representing a wide range of experiences and roles in chemistry‐related fields (Please see Figure S3 for geographic statistics of the participants). This survey aimed to evaluate the chemists' opinions on the interpretability of the UHSR model vs. traditional machine learning models. Participants provided their feedback based on their professional experience, and demographic data such as years of experience, educational background, and geographical region were collected to ensure a comprehensive analysis. A total of 101 chemists agreed to participate. One response was excluded due to an invalid answer, leaving 100 valid responses.

### 3.4 Experimental Settings and Training Details

When constructing the modular neural network, at each stage, we set the number of neurons in the hidden layers of each sub‐model to 50. Furthermore, two hidden layers were employed. To facilitate the discovery of concise equations, we leaned toward employing a relatively simple network structure, which helps to prevent overfitting to experimental noise. The activation function chosen for each sub‐model was LeakyReLU. In the first stage, the activation function of the output layer was set to a sigmoid, which ensures a standardized output within the range of 0 and 1 (Figure 3B). The batch size was set to 2048, and the Adam optimizer implemented in PyTorch was applied. The learning rate was 0.01.

To evaluate the performance and generalizability of UHSR, we employed 10‐fold cross‐validation. The full dataset was randomly partitioned into 10 equal subsets. For each fold, 9 folds were used to train the model, and the remaining fold was used for testing. This process was repeated 10 times, ensuring that each data point was used for both training and testing. Each modular neural network was trained for 1000 epochs, then the best validation checkpoint was selected for testing. We conducted the evaluation in two parts: the modular neural networks to produce retention indices, and the derived $R_{f}$ equations.

The performance of the modular neural networks was evaluated in each fold using the R‐squared coefficient ($R^{2}$) and the root mean square error (RMSE), which are defined as

$$\begin{array}{c} R^{2}=1-\frac{\sum_{i=1}^{N}{\left(y_{i}-{\hat{y}}_{i}\right)}^{2}}{\sum_{i=1}^{N}{\left(y_{i}-\bar{y}\right)}^{2}},\;\mathrm{RMSE}=\sqrt{\frac{\sum_{i=1}^{N}{\left(y_{i}-{\hat{y}}_{i}\right)}^{2}}{N}} \end{array}$$

where N is the total number of samples, $y_{i}$ and ${\hat{y}}_{i}$ represent the label and the prediction value for the i‐th sample, and $\bar{y}$ denotes the mean of the ground truth values.

The results, presented in Table S2, demonstrate consistently high $R^{2}$ values and low RMSE values across all folds, indicating the robustness of the modular neural networks. We also derived the $R_{f}$ governing equations of each fold, as shown in Table S3. Most equations share a similar structure. Although the coefficients for Ψ and ξ vary slightly across folds, but generally remain within a consistent range.

## 4 Discussion and Conclusion

*Chellenges in current approaches to explainability*. Recent efforts to establish quantitative structure‐polarity relationships primarily rely on machine learning models and the development of informative feature representations [16, 23]. While these approaches achieve high prediction accuracy, a significant limitation lies in their lack of interpretability. This lack of transparency hinders a deeper understanding of the intrinsic relationships within the data, consequently reducing the scientific impact of these models by limiting researchers' ability to trust and fully comprehend their predictions [26, 27, 28].

To address this challenge, explainable methods have been developed, broadly categorized into intrinsic and post‐hoc approaches. Post‐hoc methods, such as LIME [51] and Shapley values [52], provide insights into the importance of input variables relative to model outputs. However, these methods are limited in their ability to explain the model's internal mechanisms, offering only a partial understanding of how features interact or contribute to predictions. They essentially provide a narrow view into the “black box,” revealing feature importance without elucidating the relationships that drive the model's decisions. Conversely, intrinsic models, such as multiple linear regression or partial least squares, are inherently interpretable but often lack the accuracy required to handle complex datasets (Figure 6B).

*The UHSR framework introduced in this study offers an alternative solution*. By integrating equation‐based modeling with hierarchical insights into feature importance. Unlike traditional methods, UHSR not only identifies key features but also explains how these features interact and propagate through the hierarchical structure to influence the final output. Key to this framework are two retention indices, the solvent retention index (Ψ) and the solute retention index (ξ). These indices provide interpretable insights into the structure‐polarity relationship while enabling direct $R_{f}$ prediction through a formula‐driven approach. By leveraging only a few indices (in this case, two) closely tied to the dataset, UHSR enhances interpretability and reduces complexity compared to high‐dimensional feature representations. Moreover, the predictive accuracy of UHSR is comparable to that of existing DNN models, demonstrating that interpretability does not need to come at the cost of performance.

*Advantages of UHSR beyond interpretability*. The symbolic regression component of UHSR offers several notable advantages over traditional machine learning models, particularly for deployment in automated platforms and resource‐constrained environments. (1) *Deployment efficiency and low computational cost*. Although the training cost of UHSR is higher than standard machine learning models due to the additional step of extracting interpretable equations, its key advantage lies in deployment efficiency. Once the symbolic equations are discovered, inference becomes nearly instantaneous, requiring only a few arithmetic operations. In contrast, black‐box models like DNNs involve evaluating large numbers of parameters during inference, leading to significantly higher computational demands. This makes UHSR particularly advantageous for automated platforms, where real‐time predictions with minimal computational overhead are critical. (2) *Robustness and transferability*. Equation‐based models have fewer parameters compared to traditional machine learning models, resulting in improved robustness and transferability. These models are less prone to overfitting and can adapt to new conditions with minimal retraining. Unlike DNNs, which often require extensive fine‐tuning and retraining when applied to new scenarios, symbolic equations derived from UHSR offer cost‐effective adaptability while retaining interpretability.

*Current limitations and future directions*. Despite these strengths, the current study has several limitations, which in turn represent exciting future directions. (1) *Dataset Coverage*. First, the UHSR model is restricted to the solvent systems represented in the TLC dataset. While the five solvents included are widely used in TLC experiments and adequately represent typical solvent systems (as indicated by 82.0% of surveyed chemists), the current framework cannot address more complex eluents with additives (e.g., AcOH or ${\mathrm{NEt}}_{3}$) or solvent systems beyond those represented in the dataset. Extending the framework to such systems would require incorporating additional experimental data and retraining the model on an expanded dataset. Second, the diversity of analytes in the training set is also limited, as it predominantly consists of substituted arenes and heteroarenes, which are rigid, planar scaffolds. These analytes' retention behavior can be effectively captured by the chemically intuitive features developed in this study, such as functional group counts and a single orientation descriptor. However, this limits the model's applicability to more diverse molecular structures. For example, heterocycles without benzene rings (e.g., thiophene, pyridine) or molecules with multiple benzene rings and fused rings (e.g., naphthalene) would require the introduction of additional chemically intuitive features, such as pyridine nitrogen, thiophene sulfur, fused ring descriptors, or steroid core types. While the current framework is not yet equipped to handle these cases, its flexibility allows for adaptation to new molecular classes once the dataset is expanded. Third, the model's focus on analytes that are predominantly sp^2^‐rich. The chemically intuitive features developed in this study may not generalize well to conformationally flexible, sp^3^‐rich molecules, even if they share similar functional groups. Future work will address this limitation by enriching the dataset with diverse 3D scaffolds and developing feature sets that incorporate 3D descriptors (e.g., conformer‐ensemble statistics, dihedral angle distributions, and 3D autocorrelation functions). These efforts will help maintain the symbolic regression framework's interpretability while extending its applicability to a broader range of chemical structures.

(2) *Non‐Uniqueness of Derived Equations*. It is important to note that the retention indices and governing equations derived from the UHSR model are not unique solutions. Due to the inherent limitations of the data, different neural networks (and their corresponding equations) can achieve similar levels of accuracy (see Tables S10–S12 for details). This phenomenon is common in scientific discovery, where multiple models can explain observed phenomena equally well. For example, in reaction kinetics, different rate laws derived from experimental data often describe the same behavior. UHSR is designed to provide one interpretable solution to the problem, aiming to enhance understanding and inspire further research within the field.

(3) *Reliance on prior knowledge for feature selection and grouping*. The current implementation of the UHSR framework relies heavily on expert‐defined feature selection and grouping strategies, which were used to construct the solvent retention index (Ψ) and solute retention index (ξ). While this approach ensures that the derived equations are chemically meaningful and interpretable, it introduces a dependency on prior chemical knowledge. This limits the scalability of the framework, particularly when applied to novel chemical spaces or datasets with less defined chemical properties. Future work will explore more automated approaches to feature selection and grouping to reduce reliance on domain expertise. For instance, unsupervised learning methods such as clustering molecular descriptors could be employed to identify functional groups or suggest feature groupings in a data‐driven manner.

*Broader implications*. While this study focuses on applications in chemistry, the development of explainable frameworks has broader relevance. By improving the interpretability of machine learning models, our framework can enhance trust in predictions and foster collaboration across scientific disciplines, contributing to advancements in data‐driven discovery.

## Ethics Statement Heading

The survey conducted in the study was submitted for ethical review and received approval from the Peking University Institutional Review Board, No. IRB00001052‐2408.

## Conflicts of Interest

The authors declare no conflicts of interest.

## Supporting information




**Supporting File**: advs73626‐sup‐0001‐SuppMat.pdf.

## Data Availability

The original code for the experiments and data analysis in this work has been deposited at the website: https://github.com/SiyuLou/UnsupervisedHierarchicalSymbolicRegression

## Footnotes

1
https://github.com/MilesCranmer/PySR

2
https://github.com/menggedu/DISCOVER
