# Supplementary material for: Unsupervised Hierarchical Symbolic Regression for Interpretable Property Modeling in Complex Multi‐Variable Systems
Source: Adv Sci (Weinh). 2026 Jan 7;13(19):e21200. doi: 10.1002/advs.202521200 (PMC13045437; doi:10.1002/advs.202521200)
Supplement: Supplementary file 1 — Supporting File: advs73626‐sup‐0001‐SuppMat.pdf. [file ADVS-13-e21200-s001.pdf]

Supporting Information for

Unsupervised hierarchical symbolic regression for transparent molecular structure-polarity analysis

Siyu Lou[a,b,†] Chengchun Liu[c,†] Dongxiao Zhang[b] Yuntian Chen[b,] Fanyang Mo[c,d,e,f,*]*

† *These authors contributed equally to this work*
* *Corresponding authors*

a. School of Computer Science, Shanghai Jiao Tong University, Shanghai 200240, P.R.China
b. Ningbo Key Laboratory of Advanced Manufacturing Simulation, Eastern Institute of Technology, Ningbo 315200, P.R.China
c. School of Materials Science and Engineering, Peking University, Beijing 100871, P.R.China
d. School of Advanced Materials, Peking University Shenzhen Graduate School, Shenzhen 518500, P.R.China
e. AI for Science (AI4S)-Preferred Program, Peking University Shenzhen Graduate School, Shenzhen 518500, P.R.China
f. Guangdong Provincial Key Laboratory of Nano-Micro Materials Research, Peking University Shenzhen Graduate School, Shenzhen 518055, P.R.China
Email Address: ychen@eitech.edu.cn, fmo@pku.edu.cn

1 Supporting Information Text

1.1 Candidate equations for the R_f governing equation

We present the candidate equations for R_f governing equation, along with the corresponding R^2 and RMSE values on the test dataset as reported in the Table S1.

Table S1: Candidate equations for $R_f \sim (\Psi, \xi)$.

Equation	R^2	RMSE
$R_f = \sigma(5.15\Psi + 5.15\xi + 1.55), \quad \sigma(x) = 1/(1+e^{-x})$	0.908	0.104
$R_f = \sigma(-\Psi\xi - \xi^2 - 5.6\xi + 5.9\Psi + 1.79), \quad \sigma(x) = 1/(1+e^{-x})$	0.909	0.104
$R_f = \sigma(3.45\Psi + \frac{-9.53\xi}{\Psi - 0.95}), \quad \sigma(x) = 1/(1+e^{-x})$	0.914	0.101
$R_f = \sigma(5.36\Psi - \frac{15.81\xi}{\xi + 1.32}), \quad \sigma(x) = 1/(1+e^{-x})$	0.917	0.099
$R_f = \sigma(\frac{6.28(\Psi^2 - 1.53\xi + 0.41)}{\Psi - \xi - 0.41}), \quad \sigma(x) = 1/(1+e^{-x})$	0.918	0.098

Symbolic regression algorithms often face a trade-off between simplicity and fitting performance. Considering the noise in real-world datasets, we prioritize simpler equations, such as choosing $R_f = \sigma(5.15\Psi + 5.15\xi + 1.55)$ as the R_f governing equation, even if there is a slight loss in fitting accuracy. On one hand, concise equations are easier to understand; on the other hand, they help avoid overfitting to noise, making the model more robust.

1.2 Comparison of solute retention index with other descriptors

While numerous existing indices, such as MPI and dipole moment, are well-established and widely used, the proposed retention indices aim to provide a more tailored approach for specific applications in TLC. The primary objective of introducing these new indices is to enhance the interpretability and applicability of predictive models in the context of TLC. Traditional indices may not fully capture the nuanced interactions between solvents and solutes in TLC experiments. Our indices are specifically designed to align with the practical experience and expectations of chemists working in this area. Specifically, the

input features of the new retention indices closely match the practice of TLC experiments. For example, the input features for the solvent retention index Ψ are the volume percentages of different solvent compounds, and for the solute retention index ξ, the counts and distribution characteristics of functional groups.
Additionally, there is a significant gap in the availability of existing descriptors and methods that meet the needs of experimental chemists for rapid polarity evaluation of new compounds.

1. Semi-quantitative empirical descriptors. Indices like ET(30), due to limited data and varying experimental conditions, often provide only qualitative descriptions. We addressed this by building a high-throughput robotic platform to obtain a standardized, high-quality TLC dataset. This dataset includes 4,944 measurements of R_f values, involving 387 organic compounds and three mobile phase systems (n-hexane/ethyl acetate, ether/n-hexane, and methanol/dichloromethane), with a total of 17 different solvent compositions.

2. High-cost theoretical calculation descriptors. Descriptors such as dipole moment and MPI, while useful, face limitations in TLC experiments that require rapid decision-making for new compounds due to their high time and computational costs. Although machine learning models can quickly derive these theoretical calculation descriptors, their characterization of molecular polarity is not as profound as the solute index we propose, which is further detailed in the following experiments.

3. Other estimated descriptors derived from SMILES. Descriptors such as TPSA and LogP can be estimated through tools like RDKit or ChemDraw, but their characterization of molecular polarity is still not as comprehensive as our proposed solute index, which is shown upon in the following experiments.

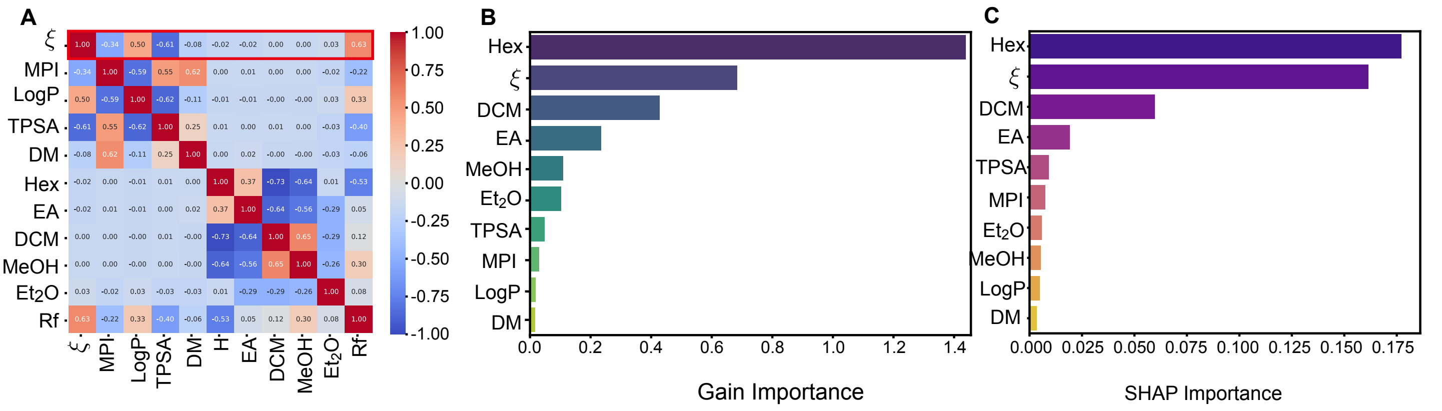

Figure S1: **(A)** Spearman Correlation Heatmap. **(B)** Feature importance by XGBoost (gain). **(C)** Feature importance by XGBoost (SHAP).

Figure S1 presents the comparison of feature importance analysis using various methods to compare the proposed solute retention index ξ with other descriptors, including TPSA, MPI, DM and LogP. Here we only investigate the indices for solute, therefore, we keep solvent features the same (5-dimensional solvent volume percentage) for all the experiments.

1. Correlation Heatmap. Figure S1A shows the Pearson correlation coefficients between different indices, including our proposed solute retention index ξ, various descriptors (*e.g.*, MPI, LogP, TPSA, DM), and R_f values. Positive correlations are indicated in red, and negative correlations are shown in blue, with the intensity representing the strength of the correlation. We can see our proposed solute retention index has the highest correlation with R_f.

2. F-Score Ranking. Figure S1B ranks the features based on their F-scores, providing a quantitative measure of each feature's significance in model performance. Higher F-scores indicate greater importance. We can see among all descriptors, our proposed solute retention ξ has the most importance.

3. SHAP Values. Figure S1C shows the mean absolute SHAP values for each feature, which quantify the contribution of each feature to the model's predictions. SHAP values offer insights into the impact of each feature across the entire dataset, highlighting their average importance. We can see among all descriptors, our proposed solute retention index ξ has the most importance.

Therefore, our proposed retention indices enable experimental chemists to evaluate molecular polarity more quickly and accurately, transitioning from qualitative to quantitative assessments.

1.3 10-fold cross validation

To evaluate the performance and generalizability of UHSR, we employed 10-fold cross validation. The full dataset was randomly partitioned into 10 equal subsets. For each fold, nine folds were used to train the model, and the remaining fold was used for testing. This process was repeated 10 times, ensuring that each data point was used for both training and testing. We conducted the evaluation in two parts: the modular neural networks to produce retention indices, and the derived R_f equations.
The performance of the modular neural networks was evaluated in each ford using the R^2 and RMSE. The results, presented in Table S2, demonstrate consistently high R^2 values and low RMSE values across all fold, indicating the robustness of the modular neural networks.

Table S2: Results of 10-Fold Cross-Validation

Split	Modular NN at stage 1		Modular NN at stage 2		Modular NN at stage 3	
	R^2	RMSE	R^2	RMSE	R^2	RMSE
0	0.9237	0.00898	0.9814	0.00500	0.9992	0.00117
1	0.9312	0.00723	0.9775	0.00100	0.9982	0.00134
2	0.9280	0.00756	0.9830	0.00170	0.9945	0.00979
3	0.9389	0.00731	0.9812	0.00350	0.9962	0.01231
4	0.9257	0.00838	0.9835	0.00264	0.9984	0.00406
5	0.9183	0.00847	0.9813	0.00349	0.9963	0.00640
6	0.9389	0.00666	0.9930	0.00122	0.9996	0.00070
7	0.9129	0.01035	0.9944	0.00086	0.9975	0.00257
8	0.9007	0.00964	0.9834	0.00214	0.9996	0.00061
9	0.9410	0.00615	0.9790	0.00190	0.9984	0.00198

We also derived the R_f governing equations of each fold, as shown in Table S3. Most equations share a similar structure. Although the coefficients for Ψ and ξ vary slightly across folds but generally remain within a consistent range.

Table S3: Results of 10-Fold Cross-Validation : R_f equation

Split	governing equations	R^2	RMSE
1	$R_f = \sigma(5.15\Psi + 5.15\xi + 1.55)$	0.908	0.104
2	$R_f = \sigma(4.02\Psi + 9.52\xi + 1.96)$	0.923	0.090
3	$R_f = \sigma(3.75\Psi + 7.62\xi + 0.62)$	0.906	0.100
4	$R_f = \sigma(2.71\Psi + 5.45\xi + 2.41)$	0.930	0.092
5	$R_f = \sigma(4.15\Psi + 6.90\xi + 1.15)$	0.909	0.101
6	$R_f = \sigma(2.06\Psi + 4.12\xi + 0.16)$	0.910	0.097
7	$R_f = \sigma(3.50\Psi + 6.01\xi - \Psi^2 - 2.10\xi^2)$	0.930	0.088
8	$R_f = \sigma(3.90\Psi + 6.54\xi + 1.29)$	0.903	0.107
9	$R_f = \sigma(4.11\Psi + 6.41\xi - 3.34\xi^2 + 2.05)$	0.844	0.123
10	$R_f = \sigma(4.61\Psi + 7.19\xi + 3.92)$	0.933	0.083

1.4 More results on R_f governing equations

***R_f* governing equation *w.r.t* physicochemical descriptors.** We used pySR with hyperparameters setup as outlined in Table S7 to derive the governing equation, yielding

$$R_f = \sigma\Big(- \text{Hex}^2 \cdot \big(1.16\text{HBA} + (\text{Hex} - \text{EA} + \text{Et}_2\text{O})^2\big) - 1.46\text{EA}^2 + 1.32\text{Et}_2\text{O}^2 - \text{HBD} + 1.40\,(0.845\text{EA} + 1)^2 + (1.36 - \text{MeOH})\left(-\frac{1.30\text{DCM}}{\text{LogP}} + 26.2\text{MeOH}\right)\Big),$$

where $\sigma(x) = 1/(1+e^{-x})$. While the equation is relatively simple, the prediction performance only reaches $R^2 = 0.664$ and RMSE= 0.194.

R_f governing equation *w.r.t* MACCS. Similarly, we derived the R_f governing equations containing MACCS features, resulting in

$$\begin{aligned} R_f = \sigma(& -1.61\text{Hex}^2 + 3\text{EA} - \text{MACCS}_{99} - \text{MACCS}_{122} + 0.778\text{MACCS}_{135} \\ & - 0.778\text{MACCS}_{140} - \text{MACCS}_{143} - \text{MACCS}_{152} - \text{MACCS}_{165} \\ & + 26.2\text{MeOH} + \text{Et}_2\text{O}^2 + \text{Et}_2\text{O} + 1.34), \end{aligned}$$

where $\sigma(x) = 1/(1+e^{-x})$. Given the inclusion of 167 MACCS features, identifying suitable variables is extremely challenging. The equation achieves a prediction performance of $R^2 = 0.734$ and RMSE= 0.172.

1.5 More results for various SR algorithms

The UHSR framework is flexible and supports multiple symbolic regression methods. In addition to the GP algorithm, we also present results from SR algorithms employing deep reinforcement learning. The open source Python package, DISCOVER, was utilized for the implementation of this method. The discovered hierarchical equation system is detailed in Table S4. The hyperparameter configurations for our implementations are detailed in Table S5.

Table S4: Hierarchical equation systems from DISCOVER.

Equation	R^2	RMSE
$R_f = \sigma\left(3.48\Psi + 3.08\xi + 1.86\right), \quad \sigma(x) = 1/(1+e^{-x})$	0.930	0.091
$\Psi = 0.73\text{EA} + 7.37\text{MeOH} - 0.27\text{DCM} + 0.51\text{Et}_2\text{O}^2 - 1.29\text{Hex}^4$	0.962	0.11
$\xi = -0.03\alpha + 0.04(e^{\alpha} - \beta)e^{\alpha} - 0.21\beta - 1.57e^{\alpha}$	0.840	0.271
$\alpha = -1.97\text{NBen} + 0.22\text{DM} - 1.49e^{\text{NBen}-\text{NBen}\times\text{DM}} + 0.12\text{NBen}\times\text{MSD}$	0.867	0.722
$\beta = 0.45\gamma_4 + 0.026(\gamma_5 - \gamma_1)^2 - 0.33\gamma_1 + 0.064\gamma_2^2 + 0.036\gamma_4^2 + 0.055\gamma_3\gamma_4 - 0.56\gamma_5 + 0.27\gamma_2$	0.793	0.975
$\gamma_1 = 1.88e^{\text{CtAmides}} - 3.93\text{CtAmides} - 2.48\text{CtAmides}^2 - 3.92\text{CtCO}_2\text{H}$	0.999	0.046
$\gamma_2 = -7.95\text{CtRNH}_2 + 9.30\text{CtOH} - 5.98\text{CtPhenol} + 1.09e^{\text{CtPhenol}-\text{CtRNH}_2} - 2.66e^{\text{CtOH}} + 2.41e^{\text{CtRNH}_2}$	0.996	0.046
$\gamma_3 = 0.93e^{\text{CtCO}_2\text{H}-\text{CtAmides}} - 3.14\text{CtAmides} - 0.014e^{\text{CtAmides}} - 2.81\text{CtCO}_2\text{H} - 0.001e^{\text{CtCO}_2\text{H}^2}$	0.991	0.203
$\gamma_4 = 1.92\text{CtCN} - 0.61\text{CtR}_2\text{CO} + 1.92\text{CtCN} + 0.32e^{\text{CtAldehyde}} + 2.34\text{CtAldehyde} - 0.095\text{CtROR}^3$ $-4.32\text{CtROR} + 0.99\text{CtROR}^2 + 0.51\text{CtF}^4 - 3.00\text{CtF}$	0.971	0.385
$\gamma_5 = 3.66\text{CtBr} + 2.43\text{CtMethyl} + 0.63\text{CtCl} - 0.64e^{\text{CtBr}} + 0.57(\text{CtCl} + 4\text{CtI})^2 - 4.42\text{CtI}$	0.935	0.780

Table S5: Hyperparameters setup for DISCOVER.

Category	Hyperparameter	Value
task	function set	[“add”, “sub”, “mul”, “div”,“n2”,“n3”,“exp”]
training	n samples	200000
	batch size	500
	epsilon	0.2
controller	learning rate	0.0025
	entropy weight	0.03
	entropy gamma	0.7

1.6 Incorporating molecular skeleton descriptor enhances predictive performance

The current dataset mainly consists of molecules with benzene-based aromatic rings, and most of which contain single rings. Due to this structural bias, we introduced a molecular skeleton descriptor(MSD) feature to enhance the accuracy and precision of R_f prediction. The MSD feature was specifically designed to provide more detailed information about the distribution of function groups within molecules. Because the distribution of functional groups within a molecule influences its properties.

In the MSD framework, the benzene ring constitutes the core structure. The descriptor values are based on the pattern of substitution on the benzene ring. The value 0 indicates the absence of a benzene ring. The value 1 denotes a mono-substituted aromatic ring. The values 2, 3, and 4 represent di-substituted aromatic rings in ortho, meta, and para positions, respectively. Values 5, 6, and 7 correspond to tri-substituted aromatic rings with 1,2,3-substitution, 1,2,4-substitution, and 1,3,5-substitution patterns, respectively. Furthermore, values 8, 9, and 10 are assigned to tetra-substituted aromatic rings with 1,2,3,4-substitution, 1,2,3,5-substitution, and 1,2,4,5-substitution patterns, respectively. Lastly, values 11 and 12 are used to denote penta-substituted and hexa-substituted aromatic rings, respectively. 12 MSD structures with the aromatic ring are illustrated in Figure S2. This descriptor provides a systematic approach to categorize the substitution patterns of benzene rings within molecular structures.

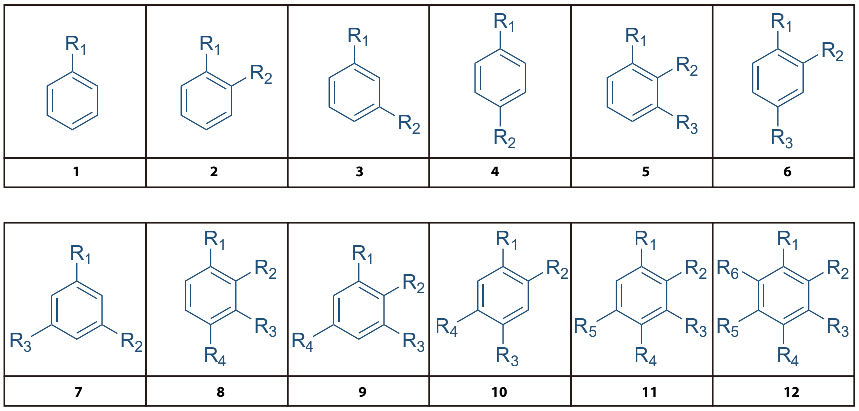

Figure S2: **Illustration of molecular skeleton descriptors.**

The derived equations are presented in Table S6.

Table S6: Optimal hierarchical equation systems corresponding to R_f and input variables (w/. MSD feature).

Equation	R^2	RMSE
$R_f = \sigma(3.48\Psi + 3.08\xi + 1.86), \quad \sigma(x) = 1/(1+e^{-x})$	0.930	0.091
$\Psi = -\mathrm{Hex} + 1.59\mathrm{EA} - 0.411\mathrm{DCM} + 11.1\mathrm{MeOH} + \mathrm{Et_2O}^2 + 0.142$	0.983	0.094
$\xi = -0.232\beta - 0.232(e^{\alpha} - 0.0531)(-2\alpha + 2\beta + 4.71)$	0.841	0.270
$\alpha = -2\mathrm{NBen}(\mathrm{DM} + 0.412) - \frac{1.33\mathrm{NBen}(\mathrm{DM}+0.412)}{\mathrm{MSD}-0.0467} - 0.743$	0.898	0.63
$\beta = -0.218\gamma_1/\gamma_2^6 + 0.413\gamma_2 + 0.435\gamma_4 - 0.435\gamma_5 + 0.0223(\gamma_3 + \gamma_4)^2 + 0.493$	0.810	0.934
$\gamma_1 = -3.09\mathrm{CtAmide} - 3.91\mathrm{CtCO_2H} + 1.87$	0.997	0.152
$\gamma_2 = -\mathrm{CtRNH_2} + 2\mathrm{CtOH} - 1.76\mathrm{CtPhenol}(1.76 - \mathrm{CtRNH_2}) + \left(-\mathrm{CtRNH_2^2} + \mathrm{CtOH} - \mathrm{CtPhenol} + 0.912\right)^2$	0.999	0.204
$\gamma_3 = \mathrm{CtNO_2}\left(-\mathrm{CtRCO_2R}^2 - 3.65\right) + 0.762$	0.961	0.413
$\gamma_4 = \mathrm{CtAldehyde}^3 - \mathrm{CtAldehyde}^2(\mathrm{CtR_2C{=}O} - \mathrm{CtF})^2 - 3\mathrm{CtAldehyde} + \mathrm{CtR_2C{=}O}^2 + 2\mathrm{CtR_2C{=}O} + 4\mathrm{CtCN} - 2\mathrm{CtF} + e^{\mathrm{CtF}-(\mathrm{CtROR}-2\mathrm{CtF})^2} - 0.746$	0.961	0.446
$\gamma_5 = (\mathrm{CtCl} + 2\mathrm{CtI})^2 + \left(\frac{\mathrm{CtBr}}{\mathrm{CtBr}-0.305} + \mathrm{CtMethyl}\right)^2$	0.931	0.805

2 Supplementary Methods

2.1 Feature sensitivity analysis

Sensitivity analysis is a widely used technique to measure the influence of each input feature on the output, by quantifying how sensitive the output is to variations in the input. When an explicit equation is available, we can directly compute this relationship mathematically, without the need for trial-and-error variations in the input. For example, to evaluate the importance of the carboxylic acid group ($\mathrm{Ct_{CO_2H}}$)

towards γ_1, we used the derived equation:

$$\gamma_1 = 5.16\text{Ct}_{\text{Am}} + 5.16\log(\text{Ct}_{\text{CO}_2\text{H}} + 0.86).$$

The sensitivity of γ_1 to $\text{Ct}_{\text{CO}_2\text{H}}$ was determined by calculating the partial derivative of γ_1 with respect to $\text{Ct}_{\text{CO}_2\text{H}}$:

$$\frac{\partial\gamma_1}{\partial\text{Ct}_{\text{CO}_2\text{H}}} = \frac{5.16}{\text{Ct}_{\text{CO}_2\text{H}} + 0.86}.$$

Our approach went beyond single-level analysis by leveraging the hierarchical structure of our equation system to propagate the sensitivity of input features through multiple intermediate stages. Specifically, input features influenced intermediate retention indices (*e.g.*, γ_1, γ_2), which were propagated through next level retention indices (such as α, β, Ψ and ξ), and ultimately to R_f. This hierarchical sensitivity analysis allowed us to assess feature importance at different levels of granularity, from individual functional groups to grouped entities such as solvent components, providing a flexible and comprehensive understanding of feature contributions.
For example, using the R_f equation ($R_f = \sigma(5.16\Psi + 5.16\xi + 1.55)$), we could calculate the importance of solvent and solute as a whole by evaluating the derivatives:

$$\frac{\partial(R_f)}{\partial\Psi} = 5.15\sigma(z)(1 - \sigma(z)),$$
$$\frac{\partial(R_f)}{\partial\xi} = 5.15\sigma(z)(1 - \sigma(z)),$$

where $z = 5.16\Psi + 5.16\xi + 1.55$. Therefore, we observed that the contributions of the solvent and solute were identical.
Another finer-grained example involved evaluating the importance of carboxylic acid group ($\text{Ct}_{\text{CO}_2\text{H}}$) towards the FG retention index β. We first considered its effect on the intermediate FG retention index, γ_1, which had a value of 5.80. This sensitivity was then propagated from γ_1 towards β, with a scaling factor of 0.028. Consequently, as shown in Figure 3E, the importance of carboxylic acid group towards the FG retention index, β, was calculated to be 0.16.
To ensure a fair comparison of global feature importance, we set all input features to their mean values in the dataset. This normalization provided a consistent and unbiased basis for comparing the contributions of different input features.

2.2 Hyperparameter configurations for pySR

We implemented the GP algorithm using the open-source Python library pySR. The hyperparameter configurations are detailed in Tables S7 and S8. To capture the higher non-linearity in the relationships between various retention indices (*e.g.*, α, β) and their corresponding input features, we increased the number of cycles per iteration. This adjustment aims to discover equations that have better fitting performance, with a trade-off of increased computation time.

2.3 Chemists' evaluation on UHSR

We provide the complete content of the survey as follows.

Survey on chemists' perspectives on ML models for molecular polarity prediction
Thank you for taking the time to participate in our survey!
Survey Description: Understanding chemists' perspectives on a new machine learning model and its application value in the field of chemistry.
Estimated Time: 5 minutes
Model Introduction: The UHSR model provides an explainable molecular polarity prediction solution, by constructing governing equations between molecular structures and their retardation factors (R_f) in thin-layer chromatography (TLC) experiments.
Data Security: The survey does not involve personal information and responses are anonymous. The data collected will be used solely for academic research.
Please read the following questions carefully and answer based on your experience and views. Each piece of feedback is valuable to our research.

1. What is your primary field of specialization?
   ☐ Organic Chemistry ☐ Inorganic Chemistry ☐ Physical Chemistry ☐ Analytical Chemistry ☐ Other (please specify)
2. How many years of experience do you have in the field of chemistry?
3. What is your gender?
   ☐ Male ☐ Female ☐ Prefer not to say
4. What is your highest level of education?
   ☐ Bachelor's Degree ☐ Master's Degree ☐ Doctoral Degree ☐ Other (please specify)
5. In which region are your research work primarily based?
   ☐ North America ☐ South America ☐ Europe ☐ Asia ☐ Africa ☐ Australia/Oceania ☐ Other (please specify)
6. Do the following five solvent compounds adequately cover the range of solvents you typically use in TLC experiments? n-Hexane (Hex), Ethyl acetate (EA), Dichloromethane (DCM), Methanol (MeOH), Diethyl ether (Et_2O).
   ☐ Completely cover ☐ Mostly cover ☐ Partially cover ☐ Do not cover ☐ I don't know
7. Artificial neural networks are black box models, while the UHSR model is an explicit equation model. Both can predict R_f values (with similar prediction accuracy). Which one do you trust more?
   Instructions: Please evaluate your level of trust in the two models. -3 represents a preference for the black-box model, 3 represents a preference for the UHSR model, and 0 represents no preference. For example, if you slightly prefer the UHSR model, please choose 1.

	-3	-2	-1	0	1	2	3	
More trust in black box models	○	○	○	○	○	○	○	More trust in the UHSR model

8. Do the retention indices provided by the UHSR model align with your expectations based on your chemical knowledge?
   Example:
   • The larger the solvent retention index value, the larger the solvent polarity.

   Solvent polarity: EA (0.73) > Hex/EA=1:1 (0.1) > Hex/EA=5:1 (-0.48) > Hex (-1.2)

   Hex: n-Hexane EA: Ethyl acetate

   • The smaller the solute retention index value, the larger the solute polarity.

   Solute polarity:

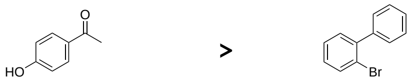

   1-(4-hydroxyphenyl)ethan-1-one(-0.74) 2-bromo-1,1'-biphenyl(0.75)

   ☐ Strongly Agree ☐ Agree ☐ Somewhat Agree ☐ Somewhat Disagree ☐ Disagree ☐ Strongly Disagree
9. Do the R_f equation derived by the UHSR model align with your expectations based on your chemical knowledge? The R_f equation is shown in the figure below. Please evaluate it based on the following instructions:
   • The R_f value is related to the polarity of the solvent and solute.
   • The greater the solvent polarity, the greater the R_f value.
   • The smaller the solute polarity, the greater the R_f value.

   larger value, larger solvent polarity (↑ Ψ)

$$R_f = \sigma(3.48\Psi + 3.08\xi + 1.86)$$

   smaller value, larger solute polarity (↓ ξ)

$$\sigma(x) = \frac{1}{1+e^{-x}}$$

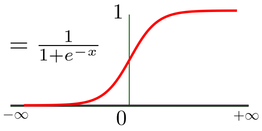

   ☐ Strongly Agree ☐ Agree ☐ Somewhat Agree ☐ Somewhat Disagree ☐ Disagree ☐ Strongly Disagree
10. Do these retention indices and the R_f equation provide new insights or knowledge that are helpful to your work?
   ☐ Strongly Agree ☐ Agree ☐ Somewhat Agree ☐ Somewhat Disagree ☐ Disagree ☐ Strongly Disagree
11. How would you rate your knowledge of artificial intelligence (AI)?

	1	2	3	4	5	6	7
I often get access to information of artificial intelligence. (1=Not at all; 7=very much)	○	○	○	○	○	○	○
I am familiar with the mechanism of artificial intelligence. (1=Not at all; 7=very much)	○	○	○	○	○	○	○
I have personal interest towards technology of artificial intelligence. (1=Not at all; 7=very much)	○	○	○	○	○	○	○

Table S7: Hyperparameters setup for discovering R_f governing equation.

Hyperparameter	Value
procs	4
population	8
population size	50
ncyclesperiteration	50
niterations	200
maxsize	50
maxdepth	10
binary operators	["+", "×", "−", "/"]
unary operators	"square"
complexity of constants	2

Table S8: Hyperparameters setup for discovering retention indices governing equation.

Hyperparameter	Value
procs	4
population	8
population size	50
ncyclesperiteration	500
niterations	200
maxsize	50
maxdepth	10
binary operators	["+", "×", "−", "/"]
unary operators	["square", "cube", "exp"]
complexity of constants	2

Table S9: Feature abbreviations for lattice parameters prediction

Abbr.	Descriptions
PE	Pauling electronegativity
Ea	Electron affinity
FC	Formal charge/valence
PCS	Pettifor chemical scale
SAR	Slater atomic radius ($\AA$)
T	Temperature ($^\circ C$)

Table S10: R_f governing equation with different random seeds when training the hierarchical neural network.

Seed	R_f governing equation	R^2
0	$R_f = \sigma(3.62\Psi + 4.82\xi + 0.573), \quad \sigma(x) = 1/(1 + e^{-x})$	0.923
1	$R_f = \sigma(5.26\Psi + 7.11\xi + 2.22), \quad \sigma(x) = 1/(1 + e^{-x})$	0.927
2	$R_f = \sigma(5.58\Psi + 8.67\xi - 0.268), \quad \sigma(x) = 1/(1 + e^{-x})$	0.929

Table S11: R_f governing equation with different neurons each layer in the hierarchical neural network.

Number of neurons/layer	R_f governing equation	R^2
50	$R_f = \sigma(3.62\Psi + 4.82\xi + 0.573), \quad \sigma(x) = 1/(1 + e^{-x})$	0.923
75	$R_f = \sigma(3.24\Psi + 5.98\xi + 0.655), \quad \sigma(x) = 1/(1 + e^{-x})$	0.916
100	$R_f = \sigma(2.68\Psi + 6.03\xi - 0.130), \quad \sigma(x) = 1/(1 + e^{-x})$	0.921

Table S12: R_f governing equation with different number of layers in the hierarchical neural network.

Number of layers	R_f governing equation	R^2
2	$R_f = \sigma(3.62\Psi + 4.82\xi + 0.573), \quad \sigma(x) = 1/(1 + e^{-x})$	0.923
3	$R_f = \sigma(3.04\Psi + 2.80\xi + 2.93), \quad \sigma(x) = 1/(1 + e^{-x})$	0.918

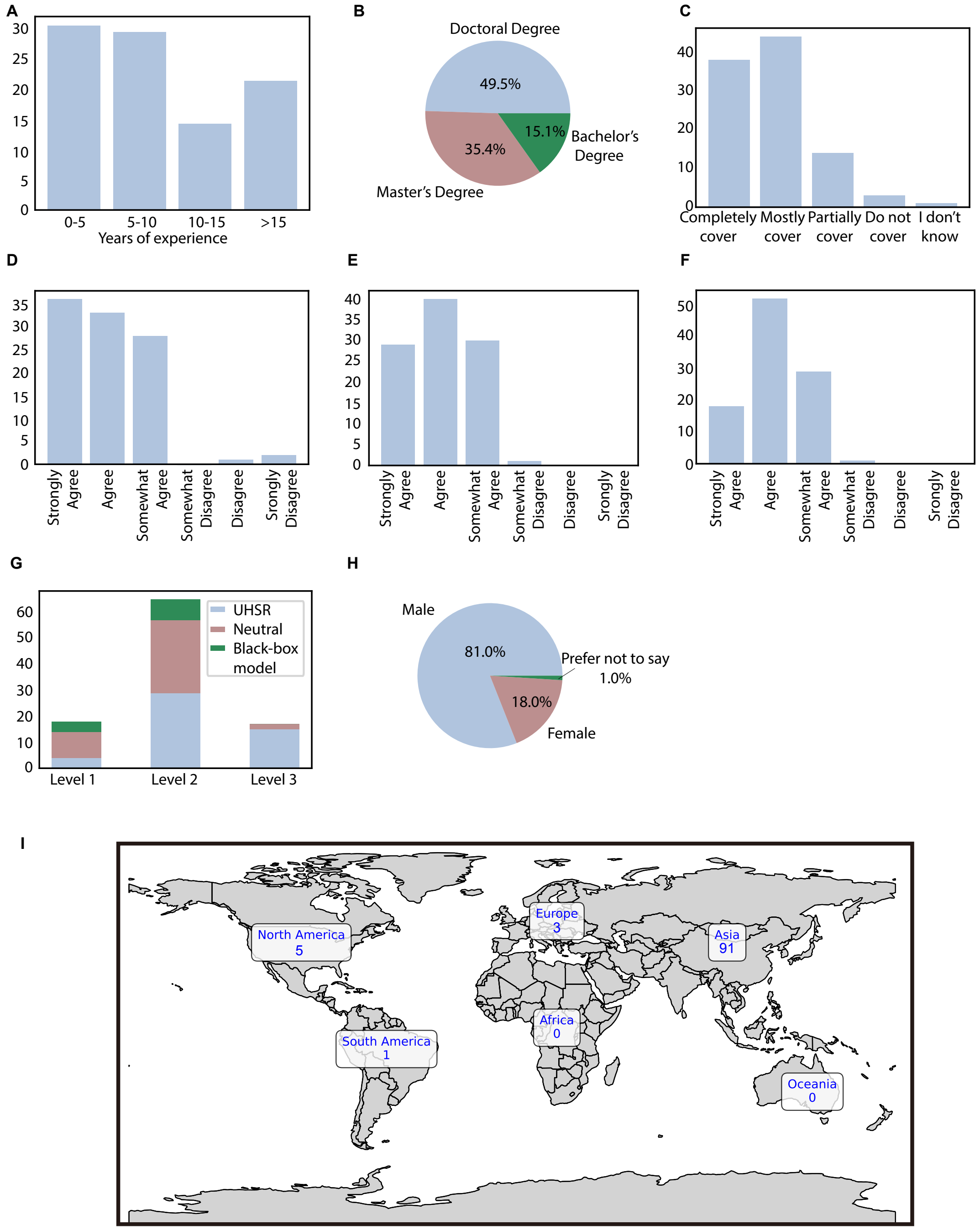

Figure S3: **Survey results statistics.** **(A)** Distribution of respondent chemists by years of experience (Q2). **(B)** Distribution of highest level of education among respondent chemists (Q4). **(C)** Responses regarding the sufficiency of solvent types used (Q6). **(D)** Chemists' expectations for the derived retention indices (Q8). **(E)** Chemists' expectations for the R_f governing equation (Q9). **(F)** Insights provided by UHiSR according to the chemists (Q10). **(G)** Chemists' trust in UHiSR versus traditional machine learning models, segmented by their level of AI knowledge (Q7, Q11). **(H)** Gender distribution of respondents (Q3). **(I)** Geographical distribution of respondents (Q5).

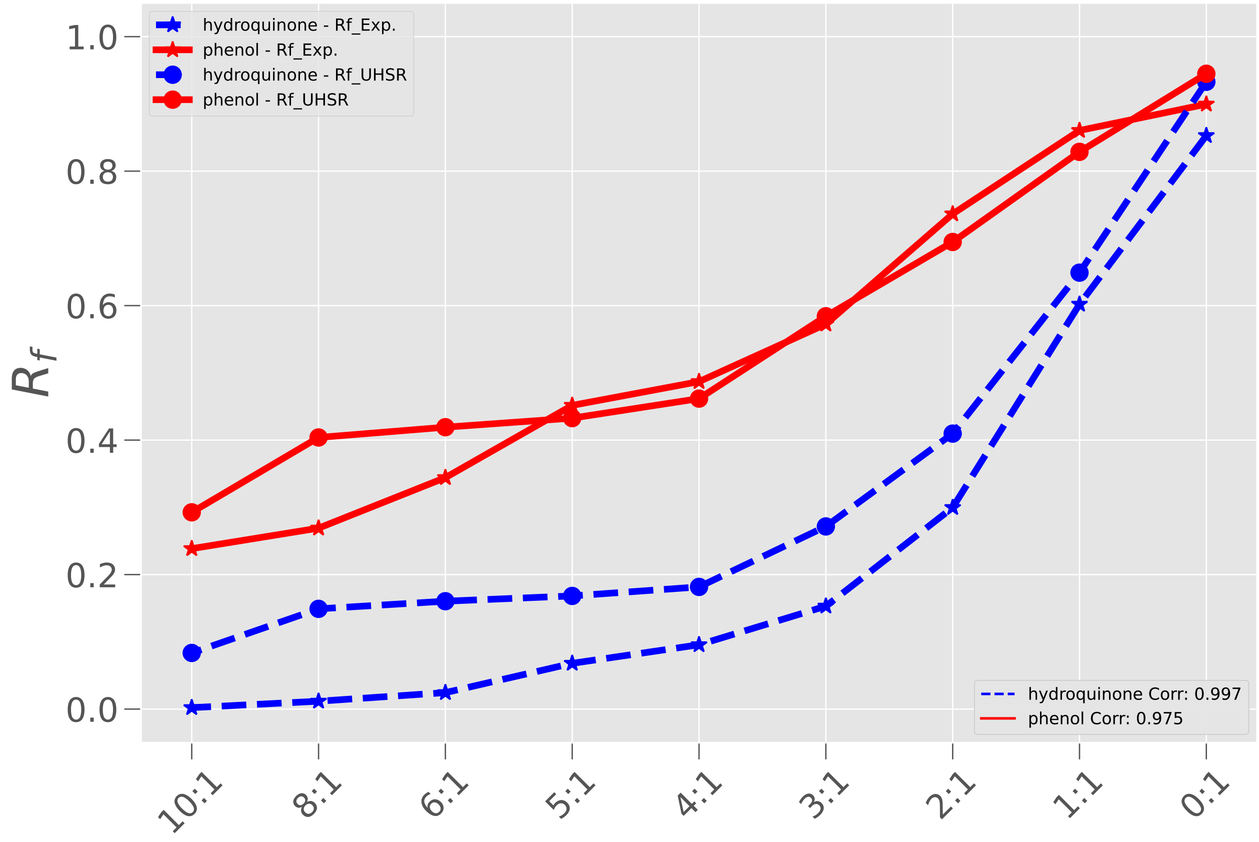

Figure S4: R_f values of hydroquinone **S1** and phenol **1** in UHSR prediction and manual TLC experiments under different solvent ratios.

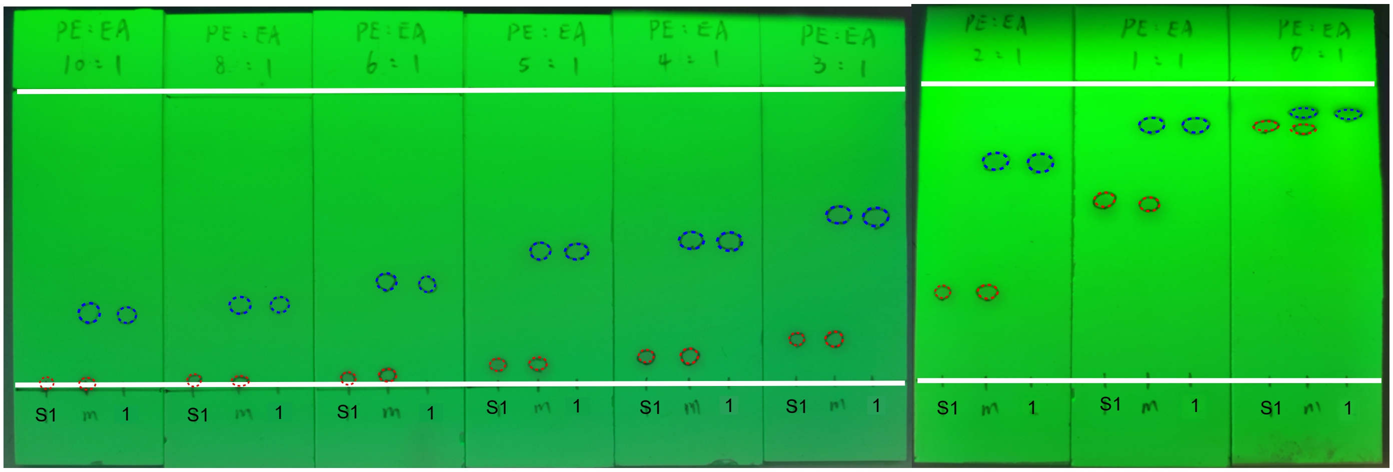

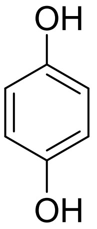
hydroquinone
S1

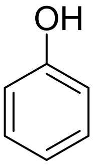
phenol
1

Figure S5: R_f values of hydroquinone **S1** and phenol **1** in manual TLC experiments under different solvent ratios. To accommodate the requirements of routine experiments, n-hexane (Hex) was replaced with petroleum ether (PE), a more commonly utilized solvent predominantly consisting of a pentane and hexane mixture in manual experiments.

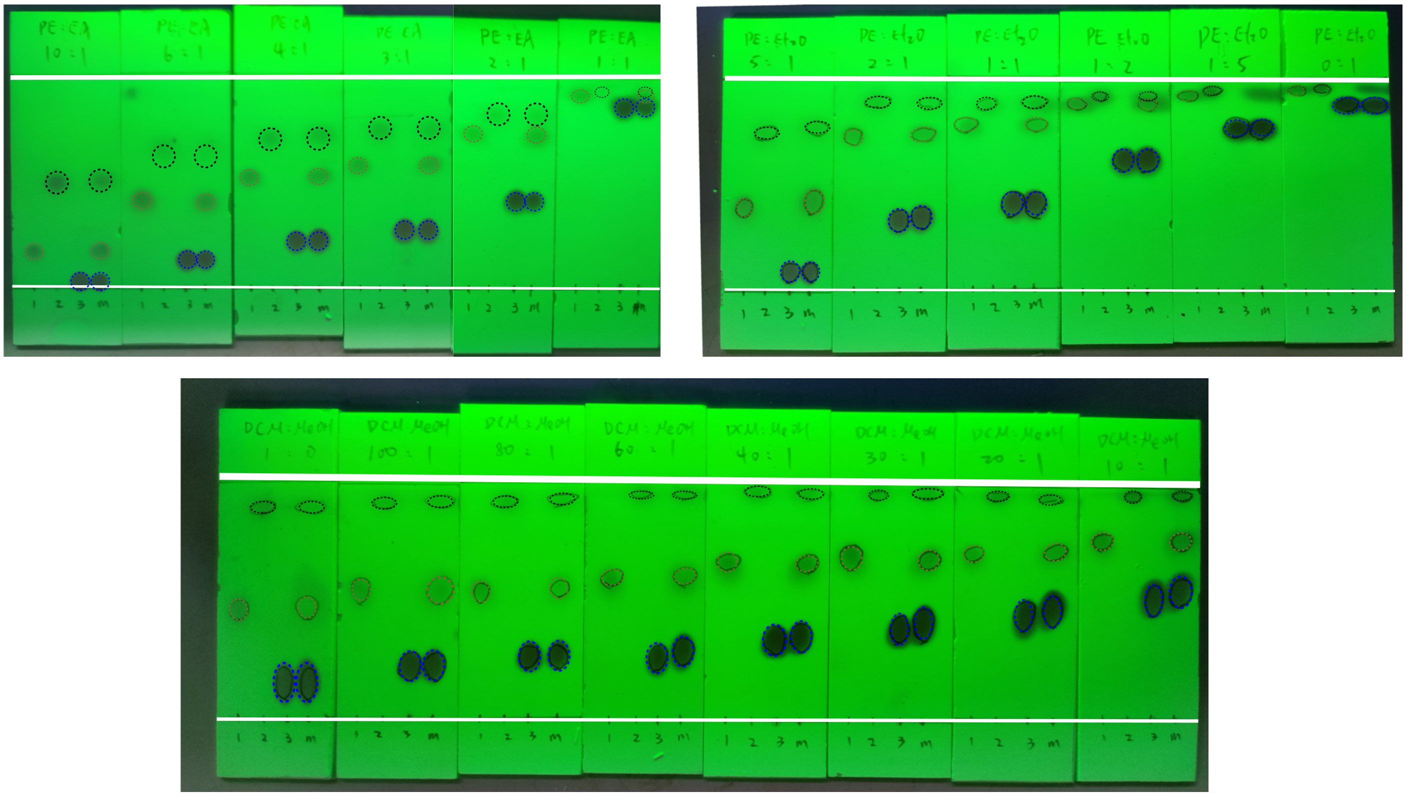

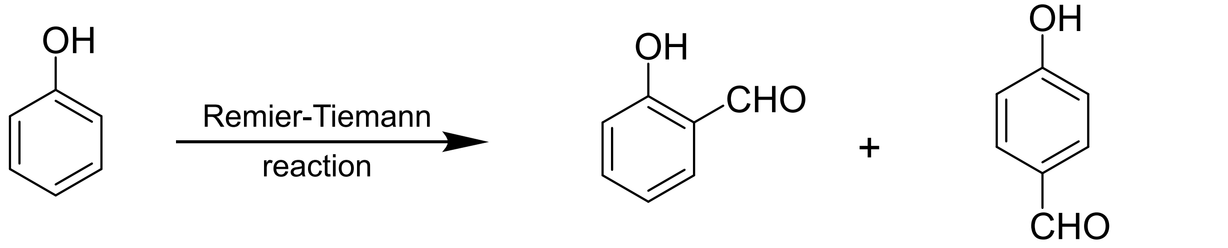

phenol **1** | *o*-hydroxybenzaldehyde **2** | *p*-hydroxybenzaldehyde **3**

Figure S6: R_f values of the product *o*-hydroxybenzaldehyde **2** and *p*-hydroxybenzaldehyde **3** of the Reimer-Tiemann reaction from phenol **1**. To accommodate the requirements of routine experiments, *n*-hexane (Hex) was substituted with petroleum ether (PE), a more commonly utilized solvent predominantly consisting of a pentane and hexane mixture.

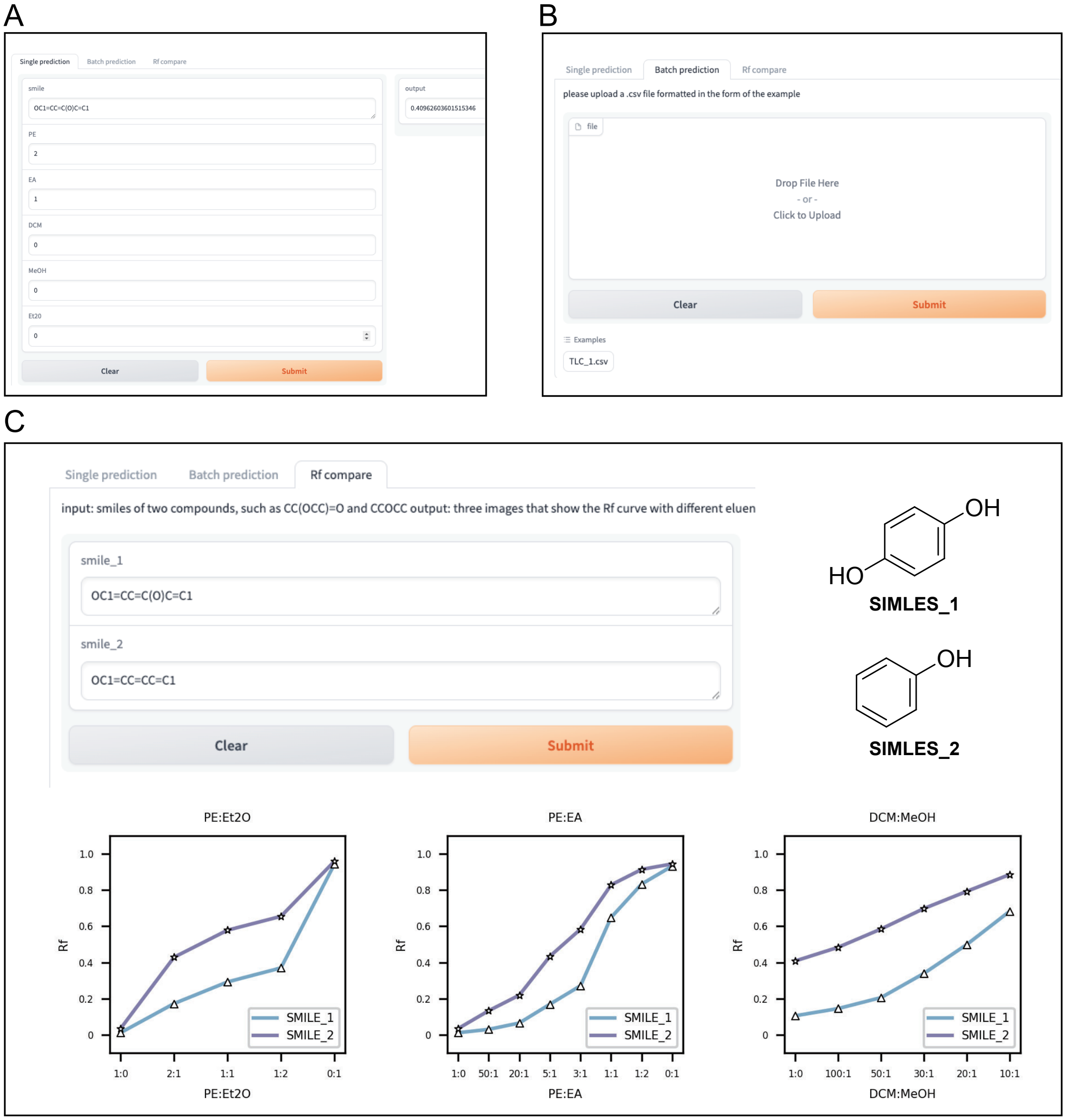

Figure S7: **Instructions for Using the R_f Value Prediction Application.** **(A)** single compound prediction. **(B)** batch prediction. **(C)** R_f compare.
